# Parieto-occipital ERP indicators of gut mechanosensation in humans

**DOI:** 10.1038/s41467-023-39058-4

**Published:** 2023-06-13

**Authors:** Ahmad Mayeli, Obada Al Zoubi, Evan J. White, Sheridan Chappelle, Rayus Kuplicki, Alexa Morton, Jaimee Bruce, Ryan Smith, Justin S. Feinstein, Jerzy Bodurka, Martin P. Paulus, Sahib S. Khalsa

**Affiliations:** 1 Laureate Institute for Brain Research, Tulsa, OK USA; 2 Department of Psychiatry, University of Pittsburgh, Pittsburgh, PA USA; 3 Harvard Medical School/McLean Hospital, Boston, MA USA; 4 Stephenson School of Biomedical Engineering, University of Oklahoma, Tulsa, OK USA; 5 Oxley College of Health Sciences, University of Tulsa, Tulsa, OK USA

**Keywords:** Sensory processing, Human behaviour

## Abstract

Understanding the neural processes governing the human gut-brain connection has been challenging due to the inaccessibility of the body’s interior. Here, we investigated neural responses to gastrointestinal sensation using a minimally invasive mechanosensory probe by quantifying brain, stomach, and perceptual responses following the ingestion of a vibrating capsule. Participants successfully perceived capsule stimulation under two vibration conditions (normal and enhanced), as evidenced by above chance accuracy scores. Perceptual accuracy improved significantly during the enhanced relative to normal stimulation, which was associated with faster stimulation detection and reduced reaction time variability. Capsule stimulation induced late neural responses in parieto-occipital electrodes near the midline. Moreover, these ‘gastric evoked potentials’ showed intensity-dependent increases in amplitude and were significantly correlated with perceptual accuracy. Our results replicated in a separate experiment, and abdominal X-ray imaging localized most capsule stimulations to the gastroduodenal segments. Combined with our prior observation that a Bayesian model is capable of estimating computational parameters of gut-brain mechanosensation, these findings highlight a unique form of enterically-focused sensory monitoring within the human brain, with implications for understanding gut feelings and gut-brain interactions in healthy and clinical populations.

## Abstract (web summary)

Understanding the neural processes governing the human gut-brain connection has been challenging. Here, the authors investigate the perceptual response and neural correlates of gastrointestinal sensation using a minimally invasive mechanosensory probe.

## Introduction

The human brain must decipher a multitude of complex signals originating from within the body to maintain homeostasis and optimally sustain life. Interoception, the process by which the nervous system senses, interprets, and integrates signals originating from within the body across conscious and nonconscious levels^1^, is a vital component of this homeostatic machinery maintaining internal stability in the face of changing environments. However, interoception remains poorly understood despite both growing scientific interest^2–5^ and the recognition that certain psychiatric^6^ and neurologic^7,8^ disorders may manifest through abnormal neural processing of interoceptive signals.

Most research on the gut-brain connection has focused on the cellular and molecular mechanisms of afferent interoceptive signal transmission in nonhuman animals^9^. For example, in the alimentary tract, rapid cell-specific peripheral sensors of osmotic balance^10^, glucose^11,12^, and mechanical stretch^13,14^ have been identified that enable organisms to quickly regulate feeding/drinking behaviors before the onset of relevant blood-level changes. Neurons in the insular cortex have been identified to play an important role in the integration of these viscerosensory signals and the adaptive estimation of upcoming needs^15,16^ – providing a focal point within the central nervous system for understanding interoceptive predictive processing. However, the brain also exerts powerful influences on the gut, and multiple descending pathways linking the stomach and brain have been identified. For example, the insular and medial prefrontal cortices send parasympathetic projections to the stomach through the vagus nerve, whereas the primary motor and somatosensory cortices send sympathetic projections to the stomach through spinal efferents^17^.

In humans, the lack of appropriate techniques for easily accessing the body’s interior has hindered the study of interoceptive processing. Some of these limitations have begun to be addressed by developing pharmacological or mechanosensory perturbations of cardiac and respiratory sensation^18,19^; yet there remains a dearth of minimally invasive probes for assessing other organ systems such as the gut. Most prior studies evaluating the conscious perception of gastrointestinal sensations have used invasive approaches involving the insertion of inflatable balloons or electrical probes into the esophagus^20,21^, stomach^22,23^, colon^24^, or rectum^25^. While such approaches have shown the ability to engage putative interoceptive cortical circuitry (i.e., insular and somatosensory cortices)^26,27^ and have revealed insights into the gut-brain axis in gastrointestinal disorders^28,29^, the invasiveness of these approaches has made it challenging to conduct such research in human participants.

We developed a minimally invasive probe targeting perceptions of the gastrointestinal system via ingestion of a vibrating capsule, and previously demonstrated that a Bayesian model can estimate the precision, prior beliefs, and learning rates associated with this form of gut stimulation^30^. In the current study, using a design inspired by signal detection theory, we combined the mechanosensory stimulation of gut signals with perceptual measurement of gut sensations and continuous recording of electroencephalogram (EEG), electrogastrogram (EGG), and other peripheral physiological signals. We identify and replicate (1) reliable signatures of gastrointestinal perception at the individual subject level and (2) differential effects in the brain based on the degree of stimulation, as measured by evoked response potentials (ERP) using EEG. We suggest that this minimally invasive approach could serve as a useful method for understanding gut-brain interactions across a variety of human health conditions.

## Results

### Demographic characteristics of original and replication datasets

40 healthy human participants (19 female, average age = 22.90 ± 4.56 years, range between 19 and 39 years, average BMI = 24.18 ± 3.03, range between 18.24 and 32.28) in the original sample completed the study and met quality assurance criteria for inclusion in the analysis. For the replication dataset, 21 healthy female volunteers (average age = 18.67 ± 4.31 years, range between 13 and 30 years, average BMI = 21.92 ± 2.75, range between 16.95 and 26.32) were included.

### Vibratory stomach stimulation to modulate gut sensation

We examined whether the noninvasive delivery of vibratory stimulation to the stomach would yield reliable signatures of gastrointestinal perception at the individual subject level. Following ingestion of the capsule, participants were instructed to press a button anytime they consciously perceived a gut sensation that they ascribed to the capsule.

#### Original experiment

##### Perceptual accuracy measures of vibration detection

**Fig. 1:**
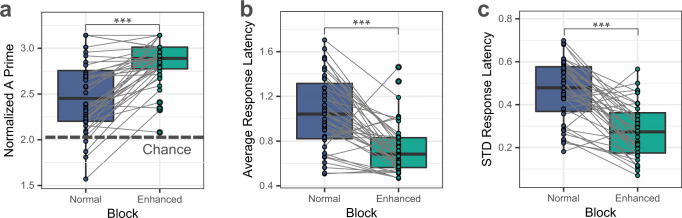
Perceptual indicators of gut sensation during vibratory gut stimulation in *n* = 40 biologically independent samples. Perceptual measures during the normal and enhanced stimulation blocks were derived based on button-presses signifying perceived sensations. Gray lines present changes in individual performance from the normal to the enhanced block. **a** Normalized A prime indicator of perceptual accuracy (dashed line shows chance performance based on binomial expansion); **b** Average response latency (in seconds); and **c** Standard deviation (STD) of the response latency (in seconds). The participants’ performance improved significantly with enhanced stimulation across all three measures, indicating the paradigm effectively induced changes in gut sensation. All paired *t*-tests (two-tailed) for perceptual accuracy measures were corrected for multiple comparisons using Bonferroni correction. ***p < 0.001. Source data are provided as a Source Data file.

The analysis of button-press responses showed that participants could successfully detect vibration stimuli under both normal and enhanced stimulation conditions, Normalized A prime (mean ± standard deviation [STD]) = 2.49 ± 0.40 and 2.84 ± 0.25, respectively. Perceptual accuracy increased significantly under enhanced versus normal stimulation (paired t-test: *p* = 4.11e-08, *t(39)* = 6.79, Cohen *d* = 1.07; Fig. 1a). Response latencies (time from capsule stimulation to button press) during normal and enhanced blocks were as follows: average response latency (mean ± STD) = 1.06 ± 0.33 seconds (normal stimulation) and 0.74 ± 0.23 seconds (enhanced stimulation); STD of response latency (mean ± STD) = 0.46 ± 0.14 (normal stimulation) and 0.28 ± 0.13 seconds (enhanced stimulation). Response latencies decreased significantly under enhanced versus normal stimulation for both the average response latency (paired *t*-test: *p* = 7.61e-07, and *t(38)* = −5.91, Cohen *d* = −0.95) and for the STD of response latency (paired t-test: *p* = 3.20e-08, and *t(38)* = −6.92, Cohen *d* = −1.11) (Fig. 1b, c). One participant did not respond to any of the normal vibration stimuli; therefore, for comparisons of response latency/STD between normal and enhanced conditions, 39 participants were included. The binomial test showed that all 40 participants performed significantly above chance (*p* < 0.05) during enhanced stimulation, whereas 5 participants performed below chance (*p* $\ge$ 0.05) during normal stimulation.

### Sex effects do not account for observed changes in gut sensation

No significant differences between the males and females were observed for any of these dependent factors, despite significant differences between normal and enhanced stimulation for all dependent variables (Table S1).

### Parieto-occipital ERP indicators of gut sensation

**Fig. 2:**
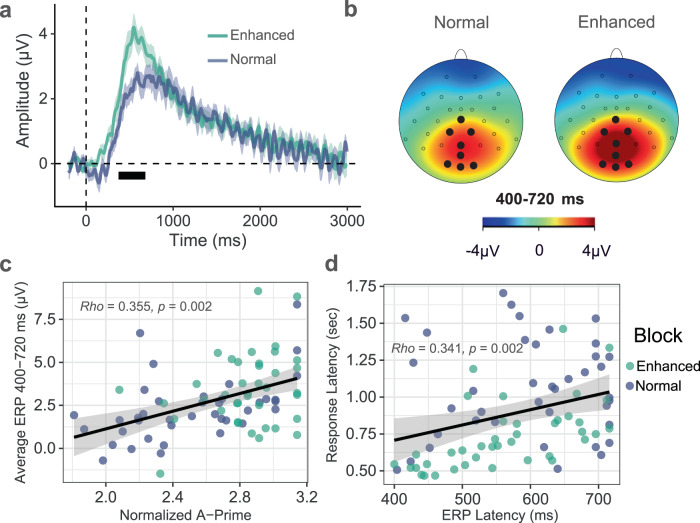
Parieto-occipital event-related potential (ERP) indicators of gut sensation during vibratory gut stimulation and their association with perceptual accuracy measures during normal and enhanced stimulation in *n* = 40 biologically independent samples. **a** The average ERP waveforms during the normal (blue) and enhanced (green) blocks for channels (Cz, CP1, CP2, Pz, POz, O1, Oz, and O2) showing the most consistent responses in the cluster-based permutation analysis. Intensity-dependent differences were identified during a late (i.e., 400–720 ms) window (marked with a horizontal black bar). Shaded areas represent the standard error of the mean for the ERP signal at each time point. Time-zero represents the earliest onset of vibratory stimulation corresponding to correctly detected vibrations, as indicated by participant button presses. The presented waveforms were calculated from the average mastoid-referenced EEG. **b** Scalp topography during the late window after the vibration onset relative to the pre-stimulus baseline for the normal and enhanced conditions. **c** The positive association between the late ERP signal strength (averaged signal among Cz, CP1, CP2, Pz, POz, O1, Oz, and O2 channels) and perceptual accuracy (normalized A prime) was significant after controlling for the condition (Spearman correlation: *ρ* = 0.354, *p* < 0.001). Average ERP amplitude data from one participant was excluded for being detected as an outlier for the enhanced condition. **d** The positive association between late ERP latency (averaged signal among Cz, CP1, CP2, Pz, POz, O1, Oz, and O2 channels) and response latency was significant after controlling for the condition (Spearman correlation: *ρ* = 0.341, *p* = 0.002). The regression lines indicate linear fits, and shaded areas correspond to the 95% confidence interval for the regressions. Source data are provided as a Source Data file.

Late positive deflections in the ERP signal emerged around 400 ms after stimulation onset, and the late positive deflections peaked around 600 ms and lasted up to 3000 ms (i.e., the duration of the vibration stimulus). These changes were maximally located over parieto-occipital electrode sites near the midline for both conditions, whereas the enhanced condition resulted in a larger late ERP amplitude than the normal condition (Figs. 2 and S1).

**Fig. 3:**
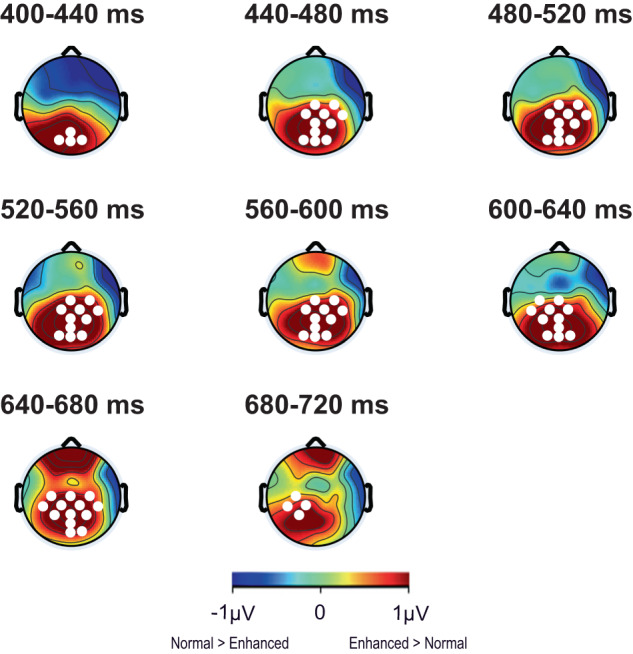
Scalp topographies for cluster-based permutation analysis on the main effect of block (normal vs. enhanced) illustrating a distribution in the late positive potential (LPP) time range of 400–720 ms. The scalp topographies for the full 3000 ms simulation period are presented in Supplementary Fig. S2. The red color bar represents higher potentials during the enhanced condition vs. normal condition, and the blue color bar represents lower potentials during the enhanced condition vs. normal condition. Electrodes that are part of clusters with *p*-values < 0.05 are depicted by white circles in the corresponding time windows. Source data are provided as a Source Data file.

We subsequently investigated the ERP difference between normal and enhanced conditions using a data-driven cluster-based permutation approach^31^. Figure 3 shows that the enhanced versus normal ERP signal differed in the late positive potential (LPP) time range (i.e., between 400 and 720 ms) in midline parieto-occipital electrodes. These results further confirm late positive deflections in both the normal and enhanced conditions.

To evaluate the potential role of motor activity (i.e., button presses) in the observed ERP signals, in a separate analysis, we examined late ERP responses to “false positive” button presses. There were no clear ERP components observed when participants responded to non-vibration stimuli (false positive button presses) or when they missed responding to the vibration (false negative) (Figs. S3 and S4 in supplement).

### Neural gut sensation indicators relate to perceptual accuracy

There was an association between the average LPP amplitude and perceptual accuracy (as measured by normalized A prime) after controlling for condition (i.e., normal/enhanced), *ρ* = 0.355, *p* = 0.002 (Fig. 2c). The correlation values for each block separately are as follows: Normal: *ρ* = 0.535, *p* < 0.001; and Enhanced: *ρ* = 0.187, *p* = 0.254 (Fig. S5A in supplement). The correlation between the response and ERP latencies was significant when controlling for condition, *ρ* = 0.341, *p* = 0.002 (Fig. 2d), and for the enhanced block separately, *ρ* = 0.683, *p* < 0.001; but non-significant for the normal block, *ρ* = 0.001, *p* = 0.998 (Fig. S5B in supplement).

### Vibratory stomach stimulation does not modulate EGG signals

**Fig. 4:**
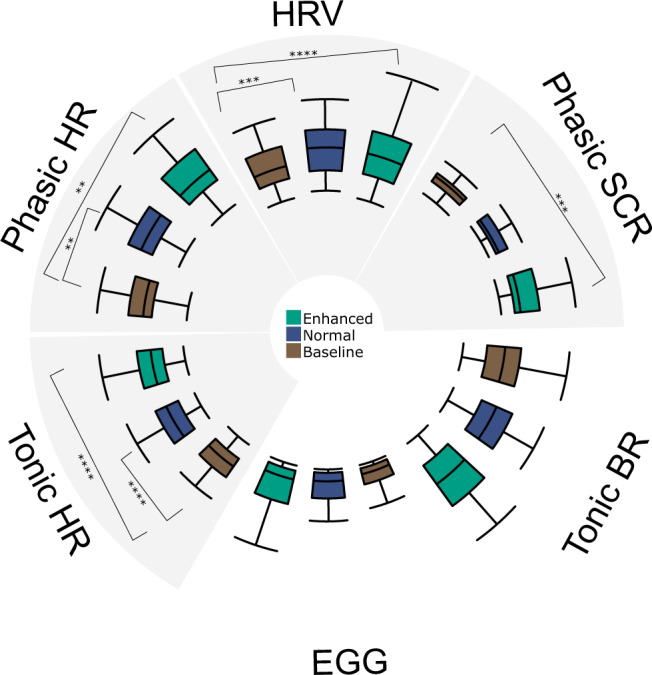
Influence of vibratory gut stimulation on peripheral physiological measures during phasic (rapid – event-related) and tonic (slow – entire block) periods. Shaded quadrants indicate signals with significant stimulation-associated changes from baseline, with brackets denoting post-hoc comparisons after Bonferroni-correction. Sample size for each block is *n* = 40 biologically independent samples unless otherwise mentioned. Top-right: phasic skin conductance response (SCR) amplitudes differed from baseline during the enhanced stimulation condition only [normal block *n* = 39]. Top-left: phasic heart rate (HR) responses differed from baseline during both the normal and enhanced stimulation conditions [enhanced block *n* = 39]. Bottom-left: tonic HR differed from baseline during both the normal and enhanced stimulation conditions. Top: tonic heart rate variability (HRV) differed from baseline during both the normal and enhanced stimulation conditions. Bottom: Total electrogastrogram (EGG) power across all physiologically relevant spectrums did not differ for each stimulation condition [enhanced block *n* = 39]. Bottom-right: estimated breathing rate (BR) responses did not differ for each stimulation condition [baseline block *n* = 39]. Linear mixed effects models were used in all comparisons. All post-hoc *p*-values were corrected for multiple comparisons using Bonferroni correction. **p < 0.01, ****p < 0.0001. Source data are provided as a Source Data file.

The EGG total power showed no significant differences between the baseline, normal, and enhanced blocks, *F(2, 75.4)* = 2.50*, p* = 0.092 (Fig. 4). With respect to the three main EGG frequency ranges (bradygastria [0.5 to 2.25] cycles per minute [cpm], normogastria [2.5–3.5 cpm], and tachygastria [3.75–9.75 cpm]), there was a significant main effect of condition *F(2305)* = 4.45*, p* < 0.05 and the main effect of frequency band *F(2305)* = 40.16*, p* < *0.0001)*, but the two-way interaction between frequency bands and condition was non-significant *F(4, 466)* = 0.62*, p* = 0.64 (Fig. S6 shows boxplots of the three EGG frequencies).

### Vibratory stomach stimulation modulates peripheral physiological signals

#### Skin conductance responses

The maximum value of phasic activity differed significantly across conditions, *F*(2,77.9) = 8.536, *p* < 0.001. Adjusted post-hoc analyses revealed significant differences between the baseline and enhanced conditions (paired *t*-test: *t*(77.1) = −4.13, *p* < 0.001, Cohen *d* = −0.92); but not for baseline and normal (paired *t*-test: *t*(77.7) = −2.08, *p* = 0.12); nor between the normal and enhanced conditions (paired *t*-test: *t*(77.7) = −2.02, *p* = 0.14; Fig. 4).

#### Cardiac responses

Tonic (i.e., basal) heart rate (HR) activity differed significantly across conditions, *F*(2,78) = 46.5, *p* < 0.0001. Bonferroni-adjusted post-hoc analyses revealed significant differences between baseline and both normal (paired t-test: *t*(78) = −7.46, *p* < 0.0001, Cohen *d* = −1.67), and enhanced conditions (paired t-test: *t*(78) = −9.02, *p* < 0.0001, Cohen *d* = −2.02), but not between the normal and enhanced conditions (paired t-test: *t*(78) = −1.56, *p* = 0.37) (Fig. 4). Phasic HR activity (i.e., fast responses specific to the 3-s vibration period) differed significantly across conditions, *F*(2,77.8) = 8.503, *p* < 0.001. Bonferroni-adjusted post-hoc analyses revealed significant differences between baseline and normal (paired *t*-test: *t*(77.1) = −3.69, *p* < 0.01, Cohen *d* = −0.83) and enhanced conditions (paired t-test *t*(77.7) = −3.43, *p* < 0.01, Cohen *d* = −0.77), while there were no differences between the normal and enhanced conditions (paired t-test: *t*(77.7) = 0.24, *p* = 1; Fig. 4). Heart rate variability (HRV), as measured through the time-domain SDNN metric, differed significantly across conditions, *F*(2,78) = 14.23, *p* < 0.0001. Bonferroni-adjusted post-hoc analyses revealed significant differences between baseline and the normal (paired *t*-test: *t*(78) = −3.81, *p* < 0.001, Cohen *d* = −0.85) and enhanced conditions (paired *t*-test: *t*(78) = −5.14, *p* < 0.0001, Cohen *d* = −1.15), while there were no differences between normal and enhanced (paired t-test: *t*(78) = −1.32, *p* = 0.57) (Fig. 4). We observed a similar pattern for HRV when using two frequency-domain metrics, the relative power of the low frequency (pLF) and high frequency (pHF) bands (Fig. S7 in the supplement).

#### Respiratory responses

Tonic breathing rate (BR) activity did not show any significant differences across conditions, *F*(2,78) = 1.82, *p* = 0.17 (Fig. 4). Descriptive statistics for all peripheral physiological signals are listed in Table S2 in the supplement.

The analysis of sex differences in the physiological data revealed no sex effect except for tonic HR responses, *F*(1, 38) = 7.808, *p* < 0.01. The post-hoc analysis for tonic HR revealed significant differences between females and males for the baseline condition (t-test: *t*(31.88) = −2.75, *p* < 0.01, Cohen *d* = −0.86), normal condition (t-test: *t*(37.77) = −2.98, *p* < 0.001, Cohen *d* = −0.94) and enhanced condition (t-test: *t*(32.46) = −2.61, *p* < 0.05, Cohen *d* = −0.82). Please refer to Table S3 in the supplement for more details.

### Vibratory capsule stimulation increases stomach/digestive sensation ratings

**Fig. 5:**
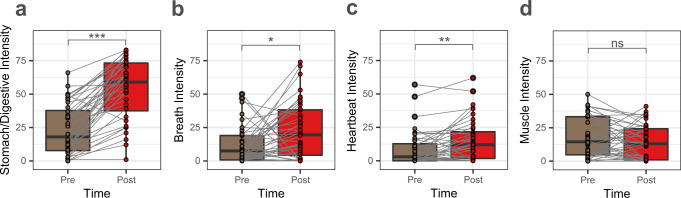
Self-reported intensity ratings of different interoceptive sensations experienced before (Pre) and during stimulation (Post) in *n* = 40 biologically independent samples. These ratings were provided retrospectively, and encompassed sensations experienced during both blocks. **a** Stomach/Digestive, **b** Breath, **c** Heartbeat, and **d** Muscle tension ratings. Stimulation-induced intensity ratings increased for stomach, breath, and heartbeat sensations. Gray lines show the change in ratings for each individual. All paired *t*-tests (two-tailed) were corrected for multiple comparisons using Bonferroni correction. *p < 0.05, **p < 0.01, ***p < 0.001; ns, not significant. Source data are provided as a Source Data file.

The LME analysis of interoceptive intensity ratings pre- and post-stimulation revealed significant differences between the rating types, *F*(3273) = 55.726, *p* < 0.001, the different time points, *F*(1273) = 48.877*, p* < 0.001, and a significant interaction between time point and rating types, *F*(3273) = 19.103*, p* < 0.001. Bonferroni-adjusted post-hoc comparison testing showed significant increases in ratings of stomach/digestive, heartbeat, and respiratory sensation intensity (paired t-test: *t*(39) = 9.861*, p* < 0.001, Cohen *d* = 1.56, for stomach/digestive intensity, *t*(39) = 3.522, *p* = 0.004, Cohen *d* = 0.56, for heartbeat intensity, and *t(39)* = 2.782*, p* = 0.033, Cohen *d* = 0.44, for respiratory intensity). There was no significant difference for muscle tension ratings (*t(39)* = −1.173, corrected *p*-value is 0.991). The estimated marginal means of pre- and post-stimulation for all four measures are presented in Table S4. These data illustrate that the magnitude of the stomach sensation intensity rating changes was larger than that for respiratory and heartbeat sensations. Figure 5 illustrates the sensation intensity ratings across the experiment. There were also significant correlations between changes in stomach/digestive, breath, and heartbeat intensity ratings (post-pre), as shown in Fig. S8. However, there were no significant correlations between ratings of muscle intensity and the other sensations. Finally, no significant differences between males and females were observed for any interoceptive intensity ratings (Table S5).

#### Gastrointestinal imaging experiment

**Fig. 6:**
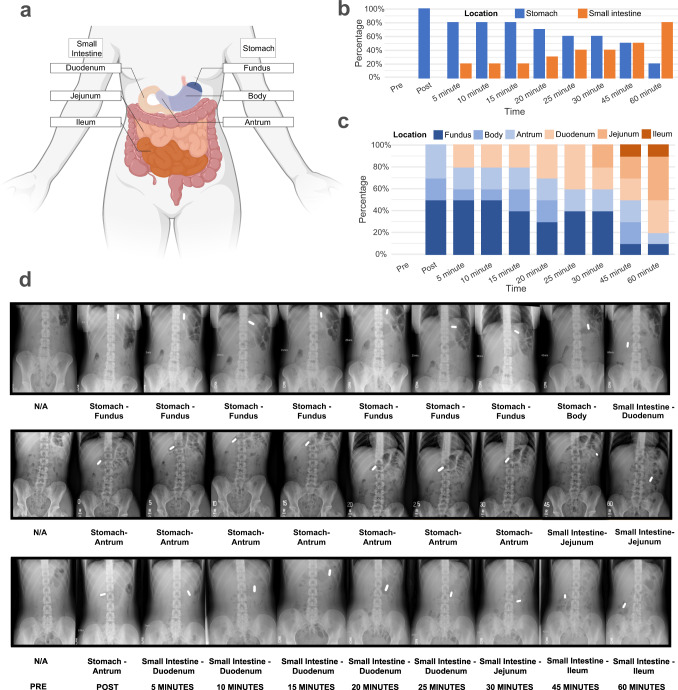
Localization of capsule stimulation to the gastroduodenal segment of the gut in *n* = 10 biologically independent samples. **a** Schematic illustration of anatomical segments of the stomach and small intestine. Intestinal contents typically transit through the stomach (fundus→body→antrum) then through the small intestine (duodenum→jejunum→ileum) before arriving in the colon (not labeled). **b** Capsule location in the stomach and small intestine as a function of time in 10 healthy individuals (5 male, 5 female) as verified by serial abdominal X-ray imaging. The capsule remained in the stomach for 60% of the participants at 30 min post ingestion. **c** Detailed illustration of capsule location in individual segments of the stomach and small intestine. The capsule remained in the stomach or duodenum for 80% of participants at 30 min post ingestion. **d** Abdominal X-rays illustrating capsule location in three participants over the course of 60 min. Each participant received 10 abdominal X-rays while lying supine. Top row: the capsule was in the stomach fundus immediately after ingestion, where it remained until 45 min. It had exited the stomach and was in the duodenum at 60 min. Middle row: the capsule was in the stomach antrum immediately after ingestion, where it remained at 30 min. It had exited the stomach and was in the jejunum at 45 min, where it remained at 60 min. Bottom row: the capsule was in the stomach antrum immediately after ingestion. At 5 min, it moved to the duodenum where it remained at 25 min. It was in the jejunum at 30 min and was in the ileum at 60 min. Source data are provided as a Source Data file.

The resulting analysis showed that most stimulations (80%) were localized to the gastroduodenal segment (Fig. 6a–c). While there was some variability in capsule location across participants, the capsule tended to remain in the same gut segment during the stimulation period in most cases (Fig. 6d).

#### Replication experiment

**Fig. 7:**
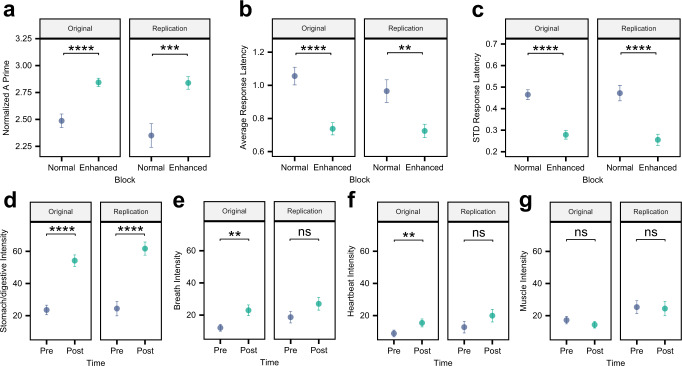
Comparisons among the original sample (*n* = 40 males and females) and replication sample (*n* = 21 females) for the capsule perceptual accuracy measures and self-reported intensity ratings. **a** Normalized A prime, **b** Average response latency, **c** Standard deviation (STD) of response latency, **d** Stomach/digestive sensation intensity, **e** Breath sensation intensity, **f** Heartbeat sensation intensity, and **g** Muscle tension sensation intensity. All paired t-tests (two-tailed) for perceptual accuracy measures and intensity ratings were corrected for multiple comparisons using Bonferroni correction. *p < 0.05, **p < 0.01, ***p < 0.001; ns, not significant. Dots represent the mean, and error bars represent the standard error around the mean. Source data are provided as a Source Data file.

Figure 7a–c illustrates the mean and standard error of the mean for the perceptual accuracy measures of vibration detection for each block and each dataset. We found similar results as the original sample in all measures, namely, higher normalized A prime in the enhanced compared to normal blocks, as well as lower response latency and standard deviation of response latency in the enhanced condition.

Figure 7d–g indicates the self-reported intensity ratings pre- and post-stimulation. Similar to the original sample, the strongest difference was observed for the stomach/digestive intensity rating in the replication dataset. Although we observed the same pattern of changes in ratings of breath and heartbeat intensity (Fig. 7e, f), these differences were not statistically significant). Finally, for muscle intensity rating, we replicated the non-significant result. Detailed comparison plots between the original and replication samples are provided for accuracy measures and self-reported intensity ratings in supplementary Figs. S9 and S10, respectively.

**Fig. 8:**
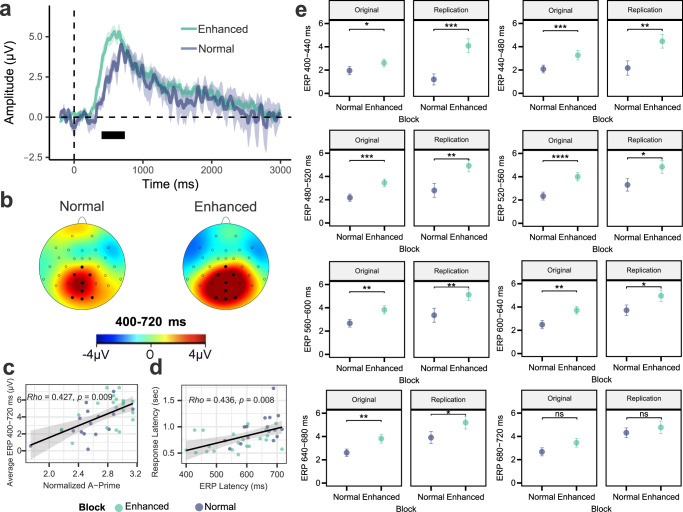
Replication of parieto-occipital event-related potential (ERP) indicators of gut sensation and their association with perceptual accuracy measures during normal and enhanced stimulation (*n* = 21 biologically independent samples). **a** The average ERP waveforms during the normal (blue) and enhanced (green) blocks for channels Cz, CP1, CP2, Pz, POz, O1, Oz, and O2. Intensity-dependent differences were examined during late (i.e., 400 to 720 ms) windows (marked with a horizontal bar). Shaded areas represent the standard error of the mean for the ERP signal at each time point. Time-zero represents the earliest onset of vibratory stimulation. The presented waveforms were calculated from the average mastoid-referenced EEG. **b** Scalp topography during the early window after the vibration onset relative to the pre-stimulus baseline for the normal and enhanced conditions. **c** The positive association between the late ERP signal strength (averaged signal among Cz, CP1, CP2, Pz, POz, O1, Oz, and O2 channels) and perceptual accuracy (normalized A prime) was significant after controlling for the condition, *ρ* = 0.441, *p* = 0.008. **d** An overall positive association between late ERP Latency (averaged signal among Cz, CP1, CP2, Pz, POz, O1, Oz, and O2 channels) and response latency was seen after controlling for the condition (*ρ* = 0.436, *p* = 0.008). **e** Comparisons among the original sample (*n* = 39; 1 participant did not detect any normal vibrations) and female replication sample (*n* = 17; 4 participant did not detect any normal vibrations) for different ERP intervals for the channels detected using the permutation approach (please refer to Fig. 4). Source data are provided as a Source Data file.

With respect to the EEG signals, similar to the original sample, we observed a LPP in midline parieto-occipital electrodes in the replication dataset (Fig. 8a, b). Positive correlations were also seen between LPP amplitude and normalized A prime (Spearman *ρ* = 0.427, *p* = 0.009; Fig. 8c), as well as between response and ERP latencies, (*ρ* = 0.436, *p* = 0.008; Fig. 8d), after controlling for condition. Significant correlations between LPP amplitude and normalized A prime were observed for the Normal (*ρ* = 0.523, *p* = 0.040) but not enhanced (*ρ* = 0.197, *p* = 0.392) blocks (Supplementary Fig. S11A). We observed a positive non-significance in the correlations between response and ERP latencies for both Normal, *ρ* = 0.422, *p* = 0.103, and Enhanced blocks, *ρ* = 0.393, *p* = 0.078 (Supplementary Fig. S11B). To test for similarities in ERP response between the original and replication sample, we selected signals from the significant channels for each time window found in the original sample (Fig. 3) and compared them across both samples. Figure 8e shows comparable findings across the samples in the form of higher ERPs in the enhanced block compared to normal in the replication sample in seven out of eight time windows. Detailed statistical comparisons, including 95% confidence interval effect size estimates between the different conditions for all parameters, are presented in Supplementary Table S6.

**Fig. 9:**
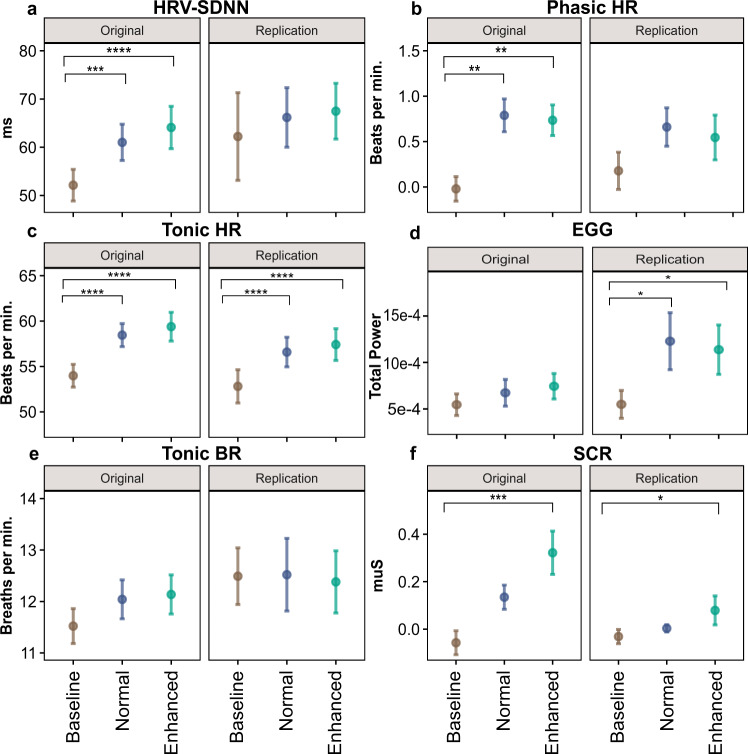
Replication of vibratory gut stimulation influence on several peripheral physiological measures (*n* = 21 biologically independent samples). Comparisons among the original sample (*n* = 40 males and females) and replication sample (*n* = 21 females) are shown for peripheral physiological measures during phasic (rapid – event-related) and tonic (slow – entire block) periods. **a** Heart rate variability (HRV) differed from baseline during both the normal and enhanced stimulation conditions [replication sample, enhanced block *n* = 20 and normal block *n* = 20]. **b** Phasic heart rate (HR) responses differed from baseline during both the enhanced stimulation conditions [original sample, enhanced block *n* = 39]. **c** Tonic HR differed from baseline during both the normal and enhanced stimulation conditions [replication sample, enhanced block *n* = 20 and normal block *n* = 20]. **d** Total electrograstrogram (EGG) power across all physiologically relevant spectrums did not differ for each stimulation condition [original sample, enhanced block *n* = 39; replication sample, enhanced block *n* = 20]. **e** Estimated breathing rate (BR) responses did not change across blocks [original sample, baseline block *n* = 39. **f** Phasic skin conductance response (SCR) amplitudes differed from baseline during the enhanced stimulation condition only [original sample, normal block *n* = 39; replication sample, enhanced block *n* = 20 and normal block *n* = 20]. **p < 0.01, ****p < 0.0001. Dots represent the mean, and error bars represent the standard error of the mean. Linear mixed effects models were used in all comparisons. All post-hoc p-values were corrected for multiple comparisons using Bonferroni correction. Source data are provided as a Source Data file.

We conducted a similar replication analysis for the psychophysiological data, including HRV, tonic HR, phasic HR phasic, tonic BR, and EGG signals. We replicated the tonic HR, tonic BR, and skin conductance response (SCR) results (Fig. 9c, e, f). For HRV-SDNN and phasic HR, similar patterns of response from baseline to normal and baseline to enhanced conditions were observed, but the differences were not statistically significant (Fig. 9a, b). However, the EGG results showed a significant change from baseline to normal and baseline to enhanced for the replication sample (Fig. 9d). A detailed statistical comparison is presented in supplementary Table S7 to assess whether the effect size of the replication analysis fell within 95% interval. Figs. S12 and S13 depict side-by-side comparisons among different peripheral physiological measures for the original sample, females of the original sample, and the replication sample.

#### Non-steroidal anti-inflammatory drug intake

Five participants in the original sample (4 females and 1 male) and 11 participants in the female replication sample reported occasional non-steroidal anti-inflammatory drug (NSAID) intake. The maximum NSAID use reported by any participant was twice per week (*n* = 1 individual), and the most common use reported by individuals was once within the prior month. Table S8 shows the results of LME testing for influences of NSAID intake in the original and replication samples. We found no effects of NSAID intake on the key variables for either sample in terms of perceptual accuracy (normalized A prime, average response latency, STD response latency), physiological data (EGG Total Power, SDNN, HR Phasic, HR Tonic, BR), or self-reported intensity rating (Stomach/Digestive, Breath, Heartbeat). There was a significant interaction between NSAID and block for phasic SCR activity in the original sample, and significant effects of medication and interaction between medication and block for self-reported muscle intensity in the replication sample, but these did not replicate across both samples.

## Discussion

In the current study, we demonstrated that a minimally invasive form of mechanosensory gastrointestinal stimulation reliably changes the perception of gut feelings. Vibratory stimulation in the gut induced evoked brain responses in midline parieto-occipital electrodes during late periods following vibration onset of the capsule. These ‘gastric evoked potentials’ (GEPs) responded to stimulation and were significantly correlated with perceptual accuracy. Furthermore, our observations of intensity-dependent increases in both perceptual accuracy measures and GEP amplitudes between the normal and enhanced forms of stimulation across two experiments indicate the reliability and effectiveness of this minimally invasive assay of gastrointestinal interoception. Mechanosensory stimulation was localized predominantly to the gastroduodenal segment and was not reliably associated with the modulation of intrinsic gastric myoelectric rhythms, though it was accompanied by reliable changes in peripheral (i.e., cardiac and electrodermal) indicators of arousal. Overall, these results highlight the presence of a distinct form of enterically-focused sensory monitoring within the human brain.

Prior functional neuroimaging investigations during mechanosensory balloon distension of the esophagus and colorectum showed activation in the primary/secondary somatosensory (SI/SII) and insular cortices^26,28,29^. We found no ERP pattern consistent with activation in these areas and instead observed midline parieto-occipital evoked potentials during mechanosensory gastroduodenal stimulation. However, these ERP findings concur with studies documenting the presence of a ‘gastric network’ in the brain^32,33^. This gastric network includes bilateral SI/SII nodes, but notably, a greater number of nodes are in the posterior cingulate sulcus, superior parieto-occipital sulcus, dorsal precuneus, retrosplenial cortex, as well as the dorsal and ventral occipital cortex. In these studies, it was predominantly the posterior midline brain regions that coactivated with the EGG signal under resting conditions, and different nodes within this network showed patterns of early and delayed changes in functional connectivity in relation to the EGG signal. Although 32-channel EEG measurements do not provide the spatial resolution necessary to optimally pinpoint the cerebral source of the observed parieto-occipital LPPs, the posterior midline brain regions observed by Rebollo et al. are located within a plausible set of brain regions for generating this result. Despite our focus on interoceptive awareness in the current study, the ERP results did not show patterns suggestive of an insular or somatosensory genesis. Instead, our findings highlight the possibility that posterior midline brain regions could play a role in interoceptive awareness of gastroduodenal sensations. This is consistent with another study based on magnetoencephalography showing an association between EGG signals and midline posterior parietal and occipital regions^34^. Using a causal interaction analysis, Richter et al. found greater evidence for information transfer from the gut to the brain than in the opposing direction (brain to the gut). There is additional human functional neuroimaging evidence of changes in posterior cingulate activity associated with gastrointestinal stimulation^35,36^. Taken together, our data support the hypothesis that gastric and intestinal interoception may be processed in posteromedial brain regions.

The posterior cingulate cortex, which is adjacent to the retrosplenial cortex, is another region that has shown selective recruitment during stimulation of the gastric fundus to elicit fullness sensations as well as pain^35^, and stimulation of this region has been associated with dissociative i.e. ‘out of body’ experience^37^. This same region has been implicated in the hierarchical mapping of autonomic nervous system input via demonstrations of abnormal activity in this region in the setting of pure autonomic failure^38^. Theoretical proposals have also pinpointed this region as a key substrate for the re-mapping of first-order body representations subserving emotion and conscious awareness in response to ongoing behavioral and environmental contexts^39^. Collectively, these studies further emphasize the role of posterior midline structures in gastrointestinal interoception.

Neither group nor individual-level analyses of the original or replication experiments provided reliable evidence that the vibrating capsule stimulation changed the frequency of stomach activity as indexed by the EGG signal. This result is important because it suggests that the afferent/ascending mechanosensory stimulation delivered by the capsule did not reliably evoke a detectable visceromotor (regulatory) gut response, at least at the systems level of measurement and sample sizes employed here. Additional studies would be needed to establish the same finding in clinical populations, particularly for those in which gastric dysrhythmias are commonly encountered^40^, and larger samples are required to detect changes in the EGG signal, such as increased slow wave amplitude, that may reflect increased gastric contractility. However, our analysis of other physiological signals revealed evidence that capsule stimulation induced changes in peripheral indicators of arousal. Specifically, phasic vibration-associated changes in heart rate and electrodermal responses were superimposed upon a tonic pattern of heightened heart rates (and altered HRV) occurring throughout both capsule stimulation conditions. These physiological findings are consistent with a broad pattern of both acute and sustained changes in autonomic activity that were induced by capsule stimulation, with a maximal shift occurring during the enhanced condition. Whether such responses reflect a general shift in autonomic reflex regulation or specific sensory or regulatory responses to gut stimulation deserves further study, particularly given arguments that arousing events can bias the attentional prioritization of information in favor of top-down processing^41^.

Minimally invasive measurement of gut sensation is an important advance given the difficulty of gaining access to the gastrointestinal system, and these findings open the possibility for future clinical disorder investigations. There are a number of poorly understood conditions in which abnormal gut sensation is a part of symptom-based care settings, including eating disorders (e.g., anorexia and bulimia nervosa)^42,43^, somatic symptom disorders^44,45^, functional neurological disorders^8,46^, and certain gastrointestinal disorders (e.g., irritable bowel syndrome (IBS), functional dyspepsia, or functional bloating^47,48^). Clinicians are often faced with a patient reporting prominent gut symptoms without a clear medical explanation, despite the understanding that these disorders involve abnormal nervous system representation of internal sensory information^6–8^. The current approach enables examinations of the relationship between gastrointestinal symptoms, illness severity, GEPs, and accuracy for detecting stomach stimulation in these clinical populations. We envision that this method could form the basis of a test assessing gut sensation in individuals with common but poorly understood gastrointestinal disorders to address major clinical issues. For example, current medical treatments for disorders of gut-brain interaction, previously known as functional gastrointestinal disorders (such as functional dyspepsia and IBS), now emphasize the adoption of a practical approach to heightened reports of gastrointestinal sensations that emphasizes the application of dietary and pharmacological treatments for central and peripheral nervous system targets as well as non-pharmacological treatments targeting psychosocial and psychotherapeutic processes^49^. Only cognitive behavioral therapy (CBT) and hypnotherapy target the hypersensitivity itself, and these behavioral treatments have shown efficacy in reducing IBS symptoms^50,51^. However, there is no way to verify perceptual sensitivity to these sensations in current clinical practice or in clinical trial settings. Adapting the capsule method, for example, to assess gut mechanosensation in disorders of gut-brain interaction before and after various forms of therapy (e.g., CBT or hypnotherapy) could be used to identify perceptual or biological mediators of successful treatment, which could be investigated as predictive/prognostic markers for prospective treatment settings. There is also the possibility of using vibratory stimulation as a form of mechanosensory biofeedback to train individuals to become more accurately perceptive of sensations from different parts of their gut (e.g., stomach versus small versus large intestine). We envision that a similar approach could be applied to psychiatric disorders such as eating disorders, for which premature gastric fullness and bloating are commonly reported but lack verifiable tests or biomarker indicators of pathology^52^.

Since the gastric and duodenal segments of the GI tract have different mechanosensory properties as well as different innervation, it will be important to evaluate how the site of stimulation impacts the psychophysiological and neural responses measured within and between individuals. This was not possible in the current study since localization information was collected during a separate visit in a subset of individuals, but this limitation could be potentially solved in future investigations. For example, approaches such as brief intermittent abdominal ultrasound might be utilized to estimate the capsule’s location (e.g., interleaved every few minutes during peri-stimulation time periods). Thus, with further testing, we envision that this approach could one day yield pathophysiological insights into functional GI and certain psychiatric disorders and potentially influence clinical decision-making. Based on the sensitivity of this measure, particularly the ability to derive robust perceptual estimates at the individual level, such studies could potentially contribute to a better accounting of the pathophysiological manifestation of symptoms (e.g., how do internally arousing stimuli contribute to the formation of the ‘symptom scaffold,’ a process whereby bodily sensations are systematically interpreted as threatening^46,53^).

A final promising future direction could be to combine this paradigm with more sophisticated computational modeling approaches that account for an individual’s prior experiences, their anticipation/prediction of future experiences, and that yield explicit parameters which can serve as metrics of each process. We envision approaches whereby specific computational parameters, such as interoceptive precision estimates, or interoceptive learning rates, can be modeled at the individual level and used to identify pathophysiologic elements of the disease process and then intervene on those elements (i.e., computational parameters) for clinical improvement. We have previously applied such approaches to address computational hypotheses about the capsule method^30^, demonstrated evidence of a failure to adapt interoceptive precision estimates for cardiorespiratory signals in psychiatric disorders^54^, and outlined the potential for computational modeling to enhance the understanding of psychiatric disorders^55^. The computational study^30^ addresses a different question focused on the hypothetical validation of active inference, a Bayesian framework related to hierarchical neural processing. The variables derived from the computational modeling of capsule induced sensation (i.e., interoceptive precision, priors, and learning rates) are distinct from those in the current study, as is the use of Bayesian modeling and Bayesian model comparison. Moreover, the prior study does not address replicability concerns or the question regarding the localization of stimulation. Thus, the current findings are distinct from the prior work. Key future applications of the current approach include the measurement of gut-brain interactions during mechanosensory gut stimulation in conditions marked by abnormal gut sensations (e.g., individuals with eating disorders or gut-brain interaction disorders such as functional dyspepsia or IBS).

In prior studies, the Vibrant capsule has been used to assess safety/tolerability in preparation for clinical trials evaluating the impact of vibration delivery in individuals with chronic constipation^56,57^. In a larger study^58^ involving active vs sham delivery of 5 capsules per week for 8 weeks, vibration sensations were reported in up to 9% of the active group vs. up to 12.5% in the sham group. However, these perception reports were obtained via retrospective symptom diaries and were related to vibration delivery to the colon (~8 h after swallowing) in individuals with chronic constipation. Given the lack of similarities with the present and previous study^30^ focusing on parameters of gut perception across very brief stimulation time scales, we find it difficult to make a meaningful comparison between them.

Studies using invasive approaches have previously identified an evoked potential during esophageal, duodenal, and anorectal stimulation^21,59^. This ‘visceral evoked potential’ was characterized by a triphasic response pattern (P1, N1, P2), usually resolving within 300 ms but differs from the signal we observed, which consisted of a monophasic peak with a delayed onset starting around 400 ms after stimulation, peaking at 600 ms, and lasting up to 3000 ms centered over the Pz and POz electrode sites. There are several potential explanations for this discrepancy. First, the studies in question used discrete painful or aversive forms of stimulation (e.g., mechanical distension or electrical shock) to induce a sensory response, whereas capsule stimulation evoked protracted nonpainful and nonaversive sensations via vibration. Second, the aforementioned studies used a single recording channel via an electrode placed at the vertex (Cz), as opposed to the 32 channels recorded across the head in the current study. We are unable to speculate on whether these studies would have seen a stronger signal localized to more posterior parietal or occipital leads if they had included a larger array of recording electrodes. Third, the invasive studies involved stimulation under passive instruction conditions, whereas the current study paired a continuous top-down (i.e., goal-directed) focus of interoceptive attention with a sustained motor response (the button press and hold), a cognitive task that presumably recruits a broader set of cortical regions. This distinction is important when considering the neural processes potentially contributing to the different phases of observed neural responses. For example, motor reaction times to passive auditory, visual, and somatosensory stimulation are typically on the order of 120 to 150 ms in duration^60^, whereas the average reaction times in the current study were about 1 second for the normal condition and 0.75 seconds for the enhanced condition (the vibration stimulus took up to a quarter second to ramp up, Fig. S14, which could have also impacted response times). While neural processing related to the motor response following perception of gastroduodenal stimulation likely contributed to some of the delays in the observed ERP signal, it is insufficient to completely explain it. Additionally, the decrease in amplitude throughout the latter two-thirds of ongoing stimulation raises the possibility that the neural encoding of this signal demonstrates typical ‘neural adaptation’, which can occur along neuronal sensory inputs and motor output pathways^61^. Thus, the observed ERPs could potentially reflect a combination of sensory, cognitive, and motor activity, and associated neural adaptations, which might help to explain why the ERPs had extended response profiles. We use the term GEP for the observed signal in part for simplicity and to acknowledge the intensity-dependent increases in amplitude in response to gastroduodenal stimulation. The prolonged time course, morphology, and scalp distribution are similar to that of the LPP^62^, which is generally thought to reflect reciprocal interactions between frontal and parieto-occipital regions, and which is sensitive to autonomic and self-reported indices of arousal^63^ and emotion regulation strategies^62^. The scalp distribution and onset of the GEP waveform could also be consistent with the P3b component, a marker of stimulus recognition^64^, although the GEP observed herein demonstrates a more protracted positivity than typical P3b studies. In sum, vibratory gastroduodenal stimulation evoked GEPs characterized by an extended positive deflection in the ERP waveform, and the amplitude of this deflection was modulated by stimulation intensity in a manner seemingly analogous to arousal modulation of visually evoked LPPs^63^. The current findings are preliminary with respect to the functional interpretation of their physiological significance, and empirical work is needed to more precisely delineate the perceptual and attentional functions that elicit and modulate its components.

The replication experiment revealed strong evidence of replication for all of the behavioral, EEG, and most subjective findings. We observed moderate evidence of replication for the psychophysiological findings via a similar pattern of findings for heart rate (tonic and phasic), heart rate variability, breathing, and skin conductance changes but not EGG changes. Specifically, while we did not observe changes in EGG activity in the original sample (*n* = 40) or the original healthy female subsample (*n* = 19), we saw evidence of changes in EGG total power in the female replication sample (*n* = 21). Given the variability in replication for the EGG signal, we are less confident in concluding that the EGG was not modified by stimulation. Further studies using larger sample sizes should be informative in this regard. The abdominal X-ray experiment revealed important evidence regarding the location of capsule stimulation, narrowing the site of most stimulations to the gastroduodenal segments of the GI tract. Although we had not expected to observe stimulation in regions outside of the stomach, mechanosensory or chemosensory stimulation of the proximal jejunum (i.e., near the duodenum) via balloon distension or capsaicin instillation, respectively, has been reported to induce sensations of pressure and abdominal discomfort across both forms of stimulation^65^. This finding suggests that the proximal portions of the small intestine, near where we localized the majority of non-gastric stimulations, contain mechanosensory receptors, and provides plausibility to the notion that participants were able to perceive capsule stimulations delivered to the duodenal segment.

This study has certain limitations. We cannot completely rule out the contribution of afferent cardio-respiratory stimulation to the observed posteromedial GEP signals, given the increased cardiac and respiratory sensation reports (for example, due to potential transmission of vibratory stimulation to the lungs through the respiratory diaphragm, which sits on top of the stomach, or due to the phasic heart rate changes observed during capsule stimulations). We would expect this degree of interference to be minimal, given the disproportionate magnitude of the effect on stomach sensation ratings and the lack of observed changes in respiratory rate. Another possibility is that attending to stomach sensations also promotes attention to cardiorespiratory sensations. One important limitation of scalp EEG recordings is that they cannot easily detect localized signals originating from deep within the cortex. Thus, we cannot conclusively exclude the possibility that the capsule stimulation also evoked changes in deep subcortical structures previously implicated in gut sensation, such as the insular cortex, thalamus, and brainstem. Studies involving positron emission tomography (PET) scanning could overcome this limitation. Functional magnetic resonance imaging (fMRI) or magnetoencephalography (MEG) would not be an option due to capsule ferromagnetic components, though, as previously mentioned, one MEG study detailed evidence of midline parieto-occipital activity linked to stomach input under resting conditions^34^. There was also potential heterogeneity in the engagement of mechanoreceptors due to unmeasured factors such as shifts in capsule orientation (i.e., vibrations could be oriented more parallel or perpendicular to the intestinal lumen over time). Several unanswered questions remain. For example, what are the molecular and cellular entities transducing the delivered stimulation? And what are the associated peripheral (presumably neural) pathways conveying the mechanosensory vibration signals to the brain? Answering these questions would likely require the application of invasive (i.e., nonhuman) studies capable of evaluating mechanotransducers at molecular levels. One candidate would be PIEZO channels^14,66^, which are heavily expressed in the stomach^67,68^ – though many other mechanical (e.g., transient receptor potential (TRP) channels) and voltage-gated ion channels are possibilities (either individually or in combination). At the cellular level, several types of mechanosensitive neurons within the enteric nervous system are known to play a role in transmitting tensile and mechanical forces, including extrinsic vagal afferent nerve endings in the upper gut and Dogiel type II neurons (spinal afferent nerve endings, which predominantly innervate the small intestine might be considered another possibility given the extension of stimulation into the proximal small intestine)^69^. Stomach stretching also elicits mechanosensory signaling that is relayed via GLP1R neurons (a vagal afferent subtype) to autonomic brainstem nuclei (nucleus tractus solitarius and area postrema)^70,71^, providing a plausible pathway by which vibratory stimulation might reach the brain. Speculation as to the subsequent trafficking of signals in the brain is beyond the scope of the current study, though others have pointed to the role of hierarchical homeostatic reflexes in the transmission^17^ and regulation^72^ of information across the brain-gut loop. These feedback loops are certainly amenable to perturbation in a variety of contexts using the gut-brain probe demonstrated here. Such perturbations would likely benefit from more precisely worded assessments and localization of abdominal sensations in future studies. Finally, while we did not find any sex effects for the observed changes in gut sensation or physiological measures, based on the low magnitude of observed sex effects, this study was likely underpowered; thus, larger studies would be required to evaluate sex differences more conclusively.

In conclusion, a minimally invasive mechanosensory probe of gastroduodenal sensation elicits intensity-dependent increases in perceptual accuracy and event-related potentials in parieto-occipital EEG leads. These changes are reliable and significantly associated with one another and are unrelated to myoelectric indices of gastric rhythm. This finding of a gastric evoked potential provides an opportunity to better understand the role of gastrointestinal symptoms in a variety of human pathological conditions and may ultimately provide insights into how gut feelings are processed by the human brain.

## Methods

### Participants

Healthy adult male and female volunteers between the ages of 18–40 years were recruited from the general Tulsa community through electronic and print advertisements for the original dataset. Eligibility was verified via the completion of structured medical and psychiatric screening evaluations. Exclusion criteria included current pregnancy or positive for drugs of abuse as defined by a urine screen during screening and during the stimulation visit, current diagnosis of psychiatric disorders, history or current diagnosis of a significant gastrointestinal disorder, gastrointestinal surgery, or other medical disorder involving respiratory, cardiovascular, renal, hepatic, biliary or endocrine disease. Prior to the capsule visit, participants underwent a screening visit during which their medical history and current medication list were assessed by a nurse trained in collecting such assessments in research settings. Medical/gastrointestinal disorders were exclusionary at the level of self-report. That is, if a participant reported a history of a gastrointestinal disorder and/or treatment for a gastrointestinal condition, they were excluded from the study. The presence of psychiatric disorders was assessed in two ways: 1) via self-report and 2) via the MINI structured clinical interview^73^ performed by a licensed mental health professional. This is a short structured diagnostic interview tailored for detecting major DSM5 psychiatric disorders.

In this study of healthy individuals, we excluded anyone taking a prescribed psychiatric medication. With respect to non-steroidal anti-inflammatory drugs (NSAIDs), we defined chronic use via use of such medications three times per week or greater. Since the use of NSAIDs is not uncommon for menstrual cramping, we did not exclude individuals using these medications on a temporary basis but tried to avoid testing individuals within two days of their last use. The study was conducted at the Laureate Institute for Brain Research, and the research protocol was approved by the Western Institutional Review Board (IRB). All participants provided written informed consent and received financial compensation for participation.

### Vibrating capsule

The vibrating capsule was developed by Vibrant Ltd and is under investigation as a non-pharmacologic therapeutic option for chronic constipation via delivery of stimulation in the colon. It consists of an orally administered non-biodegradable capsule that is wirelessly activated using an activation base unit. The Vibrant capsule is a non-significant risk device (NSR). The safety of this approach has been established in healthy human volunteers^56^ and patients with chronic constipation^57,58^.

### Masking procedure

To constrain expectancies, participants were told that two different modes of the Vibrant capsule were being evaluated and that they would be randomly assigned to one of three arms of the study: capsule mode A or B (during which the capsule would vibrate) or a placebo capsule that did not vibrate. Participants were further informed that neither they nor the experimenter would know whether any stimulations would occur. However, in actuality, every participant received a capsule that delivered vibratory stimulations, making this a single-blinded protocol. Participants were instructed to begin fasting (defined as no food or drink for 3 h prior to the study visit). We chose 3 hours of fasting based on the rationale that healthy individuals without gastrointestinal disorders who would have eaten more than 3 hours ago would be likely to have a mostly empty stomach.

### Mechanosensory stimulation

Delivery of mechanosensory stimulations to the stomach started shortly following ingestion of the Vibrant capsule. Capsule activation occurred by placing the capsule in the base unit. Shortly after activation, participants ingested the capsule with ~240 ml of water while seated in a chair. They were subsequently asked to attend to their stomach sensations while resting their eyes on a fixation cross displayed on a monitor ~60 cm away. They were instructed to use their dominant hand to press and hold a button each time they felt a sensation that they ascribed to the capsule and to release the button as soon as this sensation had ended. Stimulations began ~3 min after capsule activation in the base unit. Participants remained seated throughout the experiment in order to reduce movement artifacts in the EEG and EGG signals. They were asked to rest their non-dominant hand on their lap as well as to avoid palpating their abdomen. They were visually observed throughout the experiment by a research assistant seated behind them to verify alertness and compliance with these instructions.

In the experiment, each participant received two blocks of vibratory stimulation (normal and enhanced) in a counterbalanced order. The normal condition entailed the delivery of a standard level of mechanosensory stimulation (as developed by Vibrant) matching the level of stimulation delivered during chronic constipation trials targeting the colon. The enhanced condition entailed the delivery of an increased level of mechanosensory stimulation, which was expected to facilitate gastrointestinal perception. Each block included a total of 60 stimulations (each 3 seconds in duration), which were delivered in a pseudorandom order across a 13-min period. After a 4-min pause, the second round of 60 stimulations was delivered in pseudorandom order during a 13-min period. Thus, participants rated the presence of gastrointestinal sensations throughout a 33-min period following capsule ingestion. Due to a technical error, 3 vibrations were missing from the enhanced vibration block in the normal/enhanced sequence.

### Vibration detection

To precisely verify the vibration timing, a digital stethoscope (Thinklabs Inc.) was gently secured against the anterior surface of the lower right quadrant of the abdomen using a Tegaderm patch (15 × 20 cm). The associated signal was continuously recorded during the entire experiment and fed into the physiological recording software at a sampling rate of 1000 Hz. To identify vibrations, we developed custom analysis scripts in Matlab R2018b (Mathworks, Inc.) and used a two-step procedure to detect the onset and offset of each delivered vibration. In the first step, the script detected the vibration timings automatically using the “*findchangepts*” function in Matlab. In the second step, the timing graph for each vibration was visually inspected and adjusted if needed. Participants for whom the amplitude of vibrations could not be confidently identified using this procedure were excluded from analysis (2 individuals; 1 had a faulty capsule that did not vibrate and was also excluded). An example of the stethoscope-derived vibration waveforms for the enhanced and normal vibration conditions is shown in supplementary Fig. S14.

### Electrophysiological recordings

EEG signals were recorded continuously using a 32-channel EEG system from Brain Products GmbH, Munich, Germany. The EEG cap consisted of 32 channels, including references, arranged according to the international 10–20 system. One of these channels recorded the electrocardiogram (ECG) signal via electrode placement on the back, leaving 31 EEG signals available for analysis. The EEG signal was acquired at a sampling rate of 5000 Hz and a measurement resolution of 0.1 µV.

Besides EEG, three additional physiological measures were used in this study, including electrogastrogram (EGG), skin conductance response (SCR), and electrocardiogram (ECG). All three physiological measurements were acquired using a Biopac MP150 Acquisition Unit (Goleta, California) with a sampling frequency of 1000 Hz. Acquisition of EEG and psychophysiological measures was synchronized via a TTL pulse signal continuously fed from the EEG/button response computer to the peripheral physiological recording computer. Cutaneous EGG signals were captured via two abdominal electrodes positioned below the left costal margin and between the xiphoid process and umbilicus. The reference electrode was positioned in the right upper quadrant in line with a previous study^74^. SCRs were recorded using gel-filled electrodes attached to the thenar and hypothenar eminences of the non-dominant palm. The ECG was recorded using two electrodes positioned in a lead-II placement. All recordings were screened for physiological artifacts (e.g., motion) and analyzed offline using AcqKnowledge 4.4.2.

### EEG data processing

The pre/post-processing of EEG data was completed using BrainVision Analyzer 2 software (Brain Products GmbH, Munich, Germany). EEG data was downsampled to 250 Hz. Next, a fourth-order Butterworth (i.e., 24 dB/octave roll-off) band-rejection filter (1 Hz bandwidth) was used to remove alternating current (AC) power line noise (60 Hz). Then, a bandpass filter between 0.1 and 80 Hz (eighth order Butterworth Filter, 48 dB/octave roll-off) was utilized to remove signals unrelated to brain activity. Afterward, the infomax independent component analysis (ICA) was applied for independent component decomposition^75^ over the entire data length after excluding intervals with excessive motion-artifact by careful visual inspection. ICA was run on the data from 31 EEG channels yielding 31 independent components (ICs). The time course signal, topographic map, power spectrum density, and energy of these ICs were utilized to manually detect and remove artifactual ICs (i.e., ocular, muscle, and single-channel artifacts)^76^. In addition to these preprocessing steps, the data was segmented from the 200 milliseconds (ms) prior to the 3000 ms post-onset of each vibration for correctly detected vibrations, as indicated by participant button presses (i.e., vibrations corresponding to true positive events). Then the data were baseline corrected to the average of the 200 ms interval preceding the vibration onset. EEG data were referenced to the average of the mastoid channels (TP9 and TP10). Finally, automated procedures were used to detect bad intervals and flatlining in the data. Bad intervals were defined as any change in amplitude between data points that exceeded 50 μv or absolute fluctuations exceeding 200 µV in any 200 ms interval of the segments (i.e., −200 to 3000 ms). Flat-lining was defined as any change of <0.5 µV in a 200 ms period. Trials were excluded if they included any of these artifacts. Cluster-based permutation tests as implemented in FieldTrip^31^, a MATLAB software toolbox, were used for determining suitable time windows and electrode sites for ERP differences in normal and enhanced blocks. In summary, first, the method collects all the trials from 2 conditions in one dataset and rearranges the trials of different conditions in a random manner called a random partition. Next, it calculates the t-statistics on this random partition. Then, it repeats the last 2 steps to create a permutation distribution (the number of permutations was set to 5000 times). Using the permutation distribution, we calculated the proportion of random partitions that resulted in a larger test statistic than the observed one (i.e., the *p*-value). Since running all potential permutations is computationally too time-consuming, Monte Carlo estimation of the permutation distribution was used to randomly sample a subset of all possible permutations. To locate the spatiotemporal effect, we selected the cluster-level statistics larger than the 95th percentile (i.e., *α* = 0.05) of the permutation as the significant findings. It should be noted that in the cluster-based procedure test comparable effects over adjacent sites and time points were clustered. We adapted the script provided in^77^ for running the cluster-based permutation analysis.

### Peripheral physiological data processing

#### Electrogastrogram

The single-channel EGG recording from each subject was divided into three blocks: baseline, normal stimulation, and enhanced stimulation, based on the counterbalanced protocol. For each block, the spectral power was computed to identify the location with the largest activity in the normogastria range (2.5–3.5 cycles per min (cpm)). The spectral power analysis retained peaks of frequency in each condition for each subject. Fast Fourier Transform (FFT) from the FieldTrip toolbox^31^ was used to estimate the spectral power with a Hanning taper to reduce spectral leakage and control frequency smoothing. To further characterize the gastric rhythm, we adopted a finite impulse response (FIR) filter to filter the EGG signal into low-frequency ranges. FIR copes very well with very low-frequency filtering (as shown in ref. ^78^). Then, we applied a Hilbert transform to compute the instantaneous phase and amplitude envelope of the gastric rhythm. To further account for bad segments in the data, we adopted the artifact detection method described in ref. ^78^. This method relies on the regularity of the computed cycle durations (the standard deviation (STD) of cycle duration from the condition). More specifically, a segment was considered an artifact if the cycle length was greater than the mean ± STD of the cycle length distribution or if the cycle showed a nonmonotonic change in phase. Following the decision tree approach, any cycle with either of these conditions was considered a bad interval and excluded from the signal. The power spectral analysis was calculated again after excluding bad segments from the EGG signal, including subsequent filtering. We report the absolute power for four gastric ranges: normogastria [2.5–3.5] cpm, tachygastria [3.75–9.75] cpm, bradygastria [0.5–2.25] cpm, and total power [0.5–11] cpm (in line with ref. ^79^).

#### Heart rate

To estimate the heart rate (HR) changes for phasic stimulation (during the vibration period; 3 secs) and pre-stimulation segments (no vibration period; the preceding 3 s), the peak of the R-waves was detected using a custom peak detection algorithm in Matlab. For each block, the difference in HR between the duration of the stimulus (3 s) and the pre-stimulus (3 s) period was reported. In order to have a baseline comparison, we added 60 pseudo-events with 3-s intervals into the 30-min baseline period (after ignoring the first 2 min to allow for reaching a physiological steady-state). Additionally, we estimated the overall HR for each block by averaging HR within 60-s windows.

#### Heart rate variability

R-wave peaks were used to calculate the heart rate variability for each block. We extracted the interbeat intervals (IBIs) for each block independently. Then a custom IBI outlier detection implemented using the HRVAS Toolbox Version 2015-12-03^80^ in Matlab was used to remove abnormal IBIs. After that, the standard deviation of the normal (NN) sinus-initiated IBI (SDNN) was calculated for each block.

#### Skin conductance response

We used Continuous Deconvolution Analysis (CDA) implemented in the Ledalab Toolbox Version 3.4.9^81^ to characterize phasic changes in SCRs between stimulation and pre-stimulation segments. Specifically, we used the maximum value of phasic activity with a threshold of 0.01 muS to quantify SCRs in each segment. Prior to phasic change extraction, we downsampled the signal to 20 Hz and applied a smoothing window of 200 ms. Similar to the HR analysis, we used the same pseudo-events from the baseline period to characterize changes in SCR relative to physiological resting conditions.

#### Breathing rate

We did not use a breathing belt to minimize the delivery of skin-mediated feedback during capsule vibrations, so we estimated breathing rate (BR) from the ECG signal. To do so, we adapted a custom MATLAB script to estimate the BR using the R-R interval peaks via an autocorrelation approach to estimate the periodic maxima corresponding to integer multiples of the signal’s fundamental frequency^82^. To improve the algorithmic estimation of BR, we divided each block into 60-s windows while applying linear detrending to ECG signal resulting in 27, 13, and 13 windows for the baseline, normal, and enhanced blocks, respectively. Then, we estimated BR for each block as the average across all windows.

### Subjective rating measures

Participants completed several self-report surveys and visual analog scales indexing subjective and metacognitive experiences related to capsule stimulation, including the perceived intensity of stomach/digestive, breath, heartbeat, and muscle sensations. The scale for the intensity ratings ranged from 0 (“Not at all/None”) to 100 (“Extremely/The most I have ever felt”). Trait scales were completed during the screening visit, with the remainder of the state scales completed during the capsule visit before and after the capsule stimulation.

### Perceptual discrimination measures

We calculated measures of interoceptive accuracy for each block and participant, adopting a non-parametric signal detection analog of d′ used for conditions with low trial numbers^83,84^ as follows:

1 $$\,{A}^{{\prime} }=\frac{1}{2}+\frac{({{{TP}}}-{{{FP}}})(1+{{{TP}}}-{{{FP}}})}{4{{{TP}}}(1-{{{FP}}})}$$

where TP represents the True Positive and FP is the False positive rate. We further normalized the A′ (A prime) scores using the following equation: $2\,{{\sin }}^{-1}(\sqrt{{A}^{{\prime} }})$, resulting in values between $0$ and $\pi$. In addition, we calculated the average and the STD of the response latency for each block and each participant. Response latency was defined as the difference between the vibration onset and the participant’s button press response, indicating a perceived sensation. Each participant’s performance was further evaluated using a binomial test to check if they performed above chance during the normal and enhanced blocks, defined as $\ge$70 correct trials (out of a possible total of 120, counting the 60 normal vibration and 60 non-vibration intervals as trials) and $\ge$ 67 correct trials (out of a possible total of 114, counting the 57 enhanced vibration and 57 non-vibration intervals as trials) corresponding to an individual performance threshold statistically above chance (*p* < 0.05) according to the binomial distribution (as in ref. ^84^). One participant did not generate any button press responses during the normal block; therefore, there was no average and STD response latency available for this individual and no line connecting their performance from the normal to enhanced blocks for those measures (e.g., in Fig. 1b).

### Outlier processing

For the psychophysiological data analysis (including ERP, EGG, etc.) and perceptual accuracy measures, we removed data points that were three standard deviations (±3 SD) from the mean before running any statistical analyses. The number of outliers for each parameter is reported in Supplementary Table S9.

### Statistical analysis

Linear mixed effect models (LME) with the block (normal, enhanced) and sex (male, female) as fixed variables, subject as a random factor, and separated subject-level performance measures (normalized A′, response latency, STD response latency) and LPP as the dependent variable were conducted to investigate the effect of sex and block in each of those perception measures. Furthermore, LME analyses were performed to investigate the effect of time (pre- and post-stimulus) and sex for the subjective measure of 4 different intensities (i.e., stomach/digestive, breath, heartbeat, and muscle). Similarly, we used LME for comparing the total EGG power, SCR, tonic HR, phasic HR, HRV, and phasic BR (separately) in three different blocks (baseline, normal, enhanced). ANOVA was used to estimate the *p*-values of the block effect and emmeans package^85^ in R for running post-hoc analysis and adjusting *p*-values. We conducted a separate LME model using the three main EGG frequency ranges (bradygastria [0.5–2.25] cycles per min [cpm], normogastria [2.5–3.5 cpm], and tachygastria [3.75–9.75 cpm]) and three blocks as a fixed effect. Several paired t-tests (two-tailed) were conducted to compare the behavioral measurements between the normal and enhanced blocks. The non-parametric Spearman’s rank correlation was used to evaluate the association between neurophysiological measurements and accuracy measurements due to the non-normal distributions of such measurements. No statistical method was used to predetermine sample size. The statistical analyses were performed using R software version 4.1.0.

### Evaluation of sex differences

A linear mixed effect model was applied to test for sex differences by using block and sex as fixed effects, and the normalized A prime, average response latency, and the STD of response latency as dependent variables (applied separately for each variable) while allowing random intercept per participants. The *p*-values of the sex and block effect were calculated via ANOVA.

### Evaluation of effects due to non-steroidal anti-inflammatory drug use

To investigate the potential effect of NSAID use on the key results we categorized participants based on their reported use into those who do not use NSAIDs and who occasionally use NSAIDs (2 or less every week). We then ran a linear mixed-effect model to assess the effect of medication and its interaction with the block (i.e., normal or enhanced) for each of the following variables: perceptual accuracy (normalized A prime, average response latency, STD response latency), physiological data (SDNN, HR Phasic, HR Tonic, BR, Max Phasic, EGG Total Power), and self-reported intensity ratings (Stomach/Digestive, Breath, Heartbeat, Muscle Tension).

### Gastrointestinal imaging experiment

To provide information on the location of the capsule within the gastrointestinal tract during the delivery of stimulation, we recruited a subsample of the original cohort (10 healthy individuals, 5 males, 5 females) and paired the delivery of capsule stimulation (normal, enhanced) in a counterbalanced fashion with serial abdominal X-ray imaging over the course of 1 hour. We obtained 10 serial abdominal X-rays using a Siemens Axiom Luminos TF system. Abdominal X-ray images were collected prior to capsule ingestion, immediately after ingestion, and then 5, 10, 15, 20, 25, 30, 45, and 60 min afterwards. The time between capsule ingestion and the first post-ingestion X-ray was ~3 min; thus, the 30-min X-ray image coincided with the termination of the capsule stimulation. Given the potential impact of positional factors on gastric emptying, during the abdominal X-ray experiment, we ensured that participants were seated in an upright position on the X-ray table with the knees flexed (matching the position during the capsule experiment) at all times except when the supine position was necessitated to obtain a quality X-ray image of the abdomen (Fig. S15). To obtain each abdominal X-ray, participants briefly turned 90 degrees and then lay supine, returning immediately to the seated position upon completion of the X-ray image. Completion of each transfer and imaging process took ~1 min, and additional movements were not encouraged throughout the imaging period.

### Replication analysis experiment

In order to assess the reproducibility of our original findings (i.e., behavioral, psychophysiological, and EEG) we recruited a replication sample (*n* = 21 female participants). For this replication sample, we recruited females only, given our laboratory’s focus on understanding the neurobiology of eating disorders (i.e., anorexia nervosa, which affects females vs. males on a 10:1 basis), and to reduce potential heterogeneity in the determination of replication. We ran the same analyses on the replication sample and assessed whether the replication analysis showed similar statistical outcomes for side-by-side and direct comparisons. The p-value threshold of 0.05 was used as a red-line criterion between replication success and failure for side-by-side comparisons. For direct statistical comparisons of differences between the original female subsample and the female replication sample, we used a p-value threshold of 0.05. We further examined whether the effect size of the replication analysis fell within the 95% confidence interval (CI) of the original results.

## Supplementary information

Supplementary information

## Data Availability

Source data are provided with this paper. The data generated in this study have been deposited in the Figshare database. The data is available under accession code: 10.6084/m9.figshare.22704319. Source data are provided with this paper.

## Source data

Source Data

## Code availability

The code generated in this study has been deposited in the Github database. The code is available under accession code: https://github.com/obada-alzoubi/gut-brain-mechanosensation.
