# Supplementary material for: Parieto-occipital ERP indicators of gut mechanosensation in humans
Source: Nat Commun. 2023 Jun 13;14:3398. doi: 10.1038/s41467-023-39058-4 (PMC10264354; doi:10.1038/s41467-023-39058-4)
Supplement: Supplementary file 1 — Supplementary information [file 41467_2023_39058_MOESM1_ESM.docx]

Parieto-occipital ERP indicators of gut mechanosensation in humans

Ahmad Mayeli^1,2^*, Obada Al Zoubi^1,3^*, Evan J. White^1^, Sheridan Chappelle^1^, Rayus Kuplicki^1^, Alexa Morton^1^, Jaimee Bruce^1^, Ryan Smith^1^, Justin S. Feinstein^1^, Jerzy Bodurka^1,4^, Martin P. Paulus^1,5^, Sahib S. Khalsa^1,5^**

^1^Laureate Institute for Brain Research, Tulsa, OK, United States

^2^Department of Psychiatry, University of Pittsburgh, USA

^3^Harvard Medical School/McLean Hospital, Boston MA, United States

^4^Stephenson School of Biomedical Engineering, University of Oklahoma, Tulsa, OK, United States

^5^Oxley College of Health Sciences, University of Tulsa, Tulsa, OK, United States

***These authors contributed equally:** Ahmad Mayeli and Obada Al Zoubi

****Correspondence:**Sahib S. Khalsa MD, PhD

6655 S Yale Ave

Tulsa, OK 74136-3326

Phone: 918 502 5743
[skhalsa@laureateinstitute.org](mailto:skhalsa@laureateinstitute.org)

**This PDF file includes:**

Figures S1 to S17

Tables S1 to S10

**Table S1:** Linear mixed effect model results comparing the Normalized A Prime, Average Response Latency, and the Standard Deviation (STD) of Response Latency (as dependent variables applied separately), with Sex and Block (i.e., normal and enhanced) as fixed factors. Source data are provided as a Source Data file.

|  | **Normalized A Prime** | | **Average Response Latency** | | **STD Response Latency** | |
| --- | --- | --- | --- | --- | --- | --- |
|  | **F-Value** | **P-Value** | **F-Value** | **P-Value** | **F-Value** | **P-Value** |
| **Sex** | 0.051 | 0.822 | 0.025 | 0.876 | 0.220 | 0.642 |
| **Block** | 45.150 | < 0.001 | 30.756 | < 0.001 | 44.520 | < 0.001 |
| **Sex*Block** | 0.722 | 0.401 | 0.140 | 0.711 | 0.041 | 0.840 |

**Fig. S1:** ERP waveforms for channels Cz, CP1, CP2, Pz, POz, O1, Oz, and O2 channels during the normal (blue) and enhanced (green) blocks. Intensity-dependent differences were observed in all channels from 400 to 720 millisecond (ms, marked with dashed lines). Shaded areas represent the standard error of the mean for the ERP signal at each time point. Time-zero represents the onset of vibration stimulus. The presented waveforms were calculated from the average mastoid-referenced EEG. Late positive windows (i.e., 400 to 720 ms) identified by the cluster permutation analysis as showing stimulation are marked with the horizontal black bar. Source data are provided as a Source Data file.


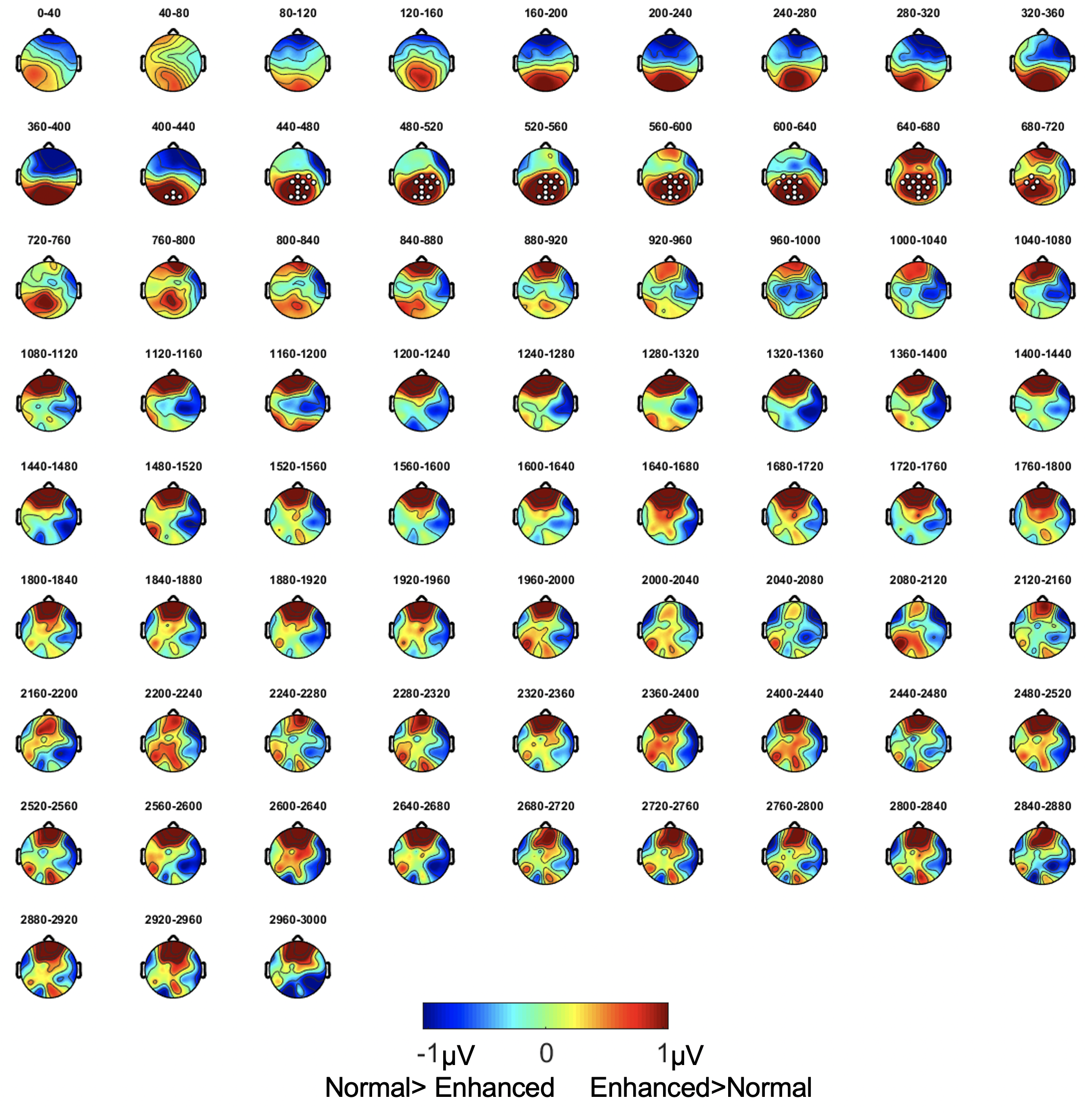


**Fig. S2:** Scalp topographies for the normal vs. enhanced ERP across the entire vibratory stimulation time range of 0 to 3000 milliseconds (ms). The red color bar represents higher potentials during the enhanced vs. normal condition, and the blue color bar represents higher potentials during the normal vs. enhanced condition. Electrodes that are part of clusters with p-values less than 0.05 are shown with white circles in the corresponding time windows, from 400 to 720 ms. Source data are provided as a Source Data file.

**Fig. S3:** Scalp topographies for false positive button presses for the late ERP response windows relative to the pre-stimulus baseline for the normal (**A**) and enhanced (**B**) blocks. The number of false positive trials was as follows: n (mean ± STD) = 2.47 ± 3.05 (normal stimulation) and 3.74 ± 3.66 (enhanced stimulation). Due to the small number of trials, these results should be interpreted with caution. Source data are provided as a Source Data file.

**Fig. S4:** Scalp topographies for false negative button presses for the ERP response windows relative to the pre-stimulus baseline for the normal (**A**) and enhanced (**B**) blocks. The number of false negative trials during normal and enhanced blocks were as follows: n (mean ± STD) = 21.25 ± 19.39 (normal stimulation) and 4.95 ± 9.34 (enhanced stimulation). Due to the small number of trials, these results should be interpreted with caution. Source data are provided as a Source Data file.

**Fig. S5: A)** The positive association between the late ERP signal strength (averaged signal among Cz, CP1, CP2, Pz, POz, O1, Oz, and O2 channels) and perceptual accuracy (normalized A prime) was significant for the normal block (Spearman correlation: *Rho* = 0.535, *p* < 0.001), but not for the enhanced block (Spearman correlation: *Rho* = 0.187, *p* = 0.254). Data from one participant was excluded for being detected as an outlier from the enhanced condition in average ERP amplitude. **B)** The positive association between late ERP Latency (averaged signal among Cz, CP1, CP2, Pz, POz, O1, Oz, and O2 channels) and response latency was significant for the enhanced block (Spearman correlation: *Rho* = 0.683, *p* < 0.001), but not for the normal block (Spearman correlation: *Rho* = 0.001, *p* = 0.998). Shaded areas correspond to the 95% confidence interval for the regressions. Source data are provided as a Source Data file.


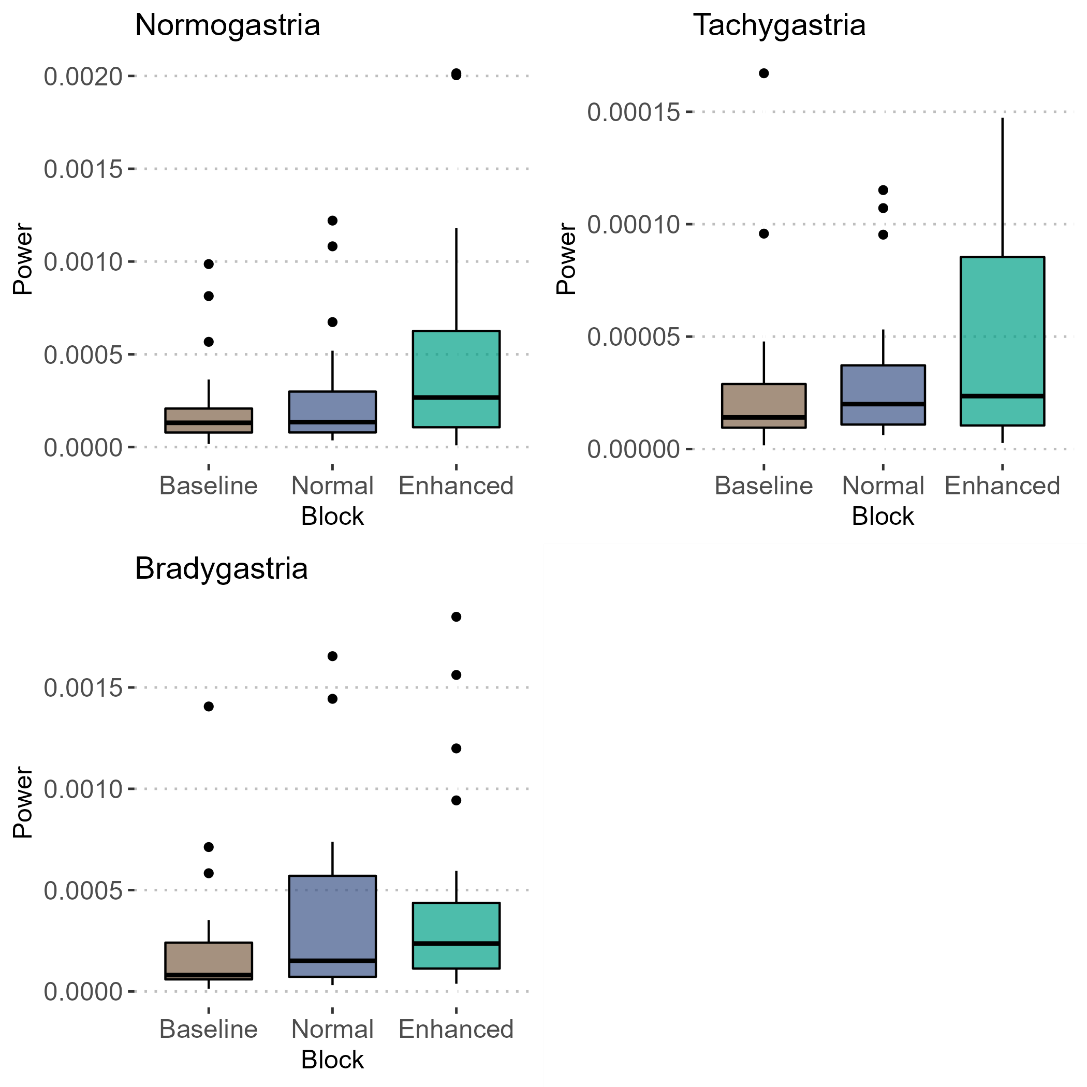


**Fig. S6:** EGG power within the Bradygastria (0.5 –2.25 cycles per minute, cpm), Normogastria (2.5 to 3.5 cpm), and Tachygastria (3.75 to 9.75 cpm) frequency spectrums. There were no statistically significant differences in power observed for each frequency spectrum across the stimulation conditions. Horizonal lines in the boxplots represent median values. Sample size (n=40) for each block except for Bradygastria-Normal (n=39), Normogastria-Normal (n=39), Normogastria-Enhanced (n=39) and Tachygastria-Enhanced (n=37). Source data are provided as a Source Data file.


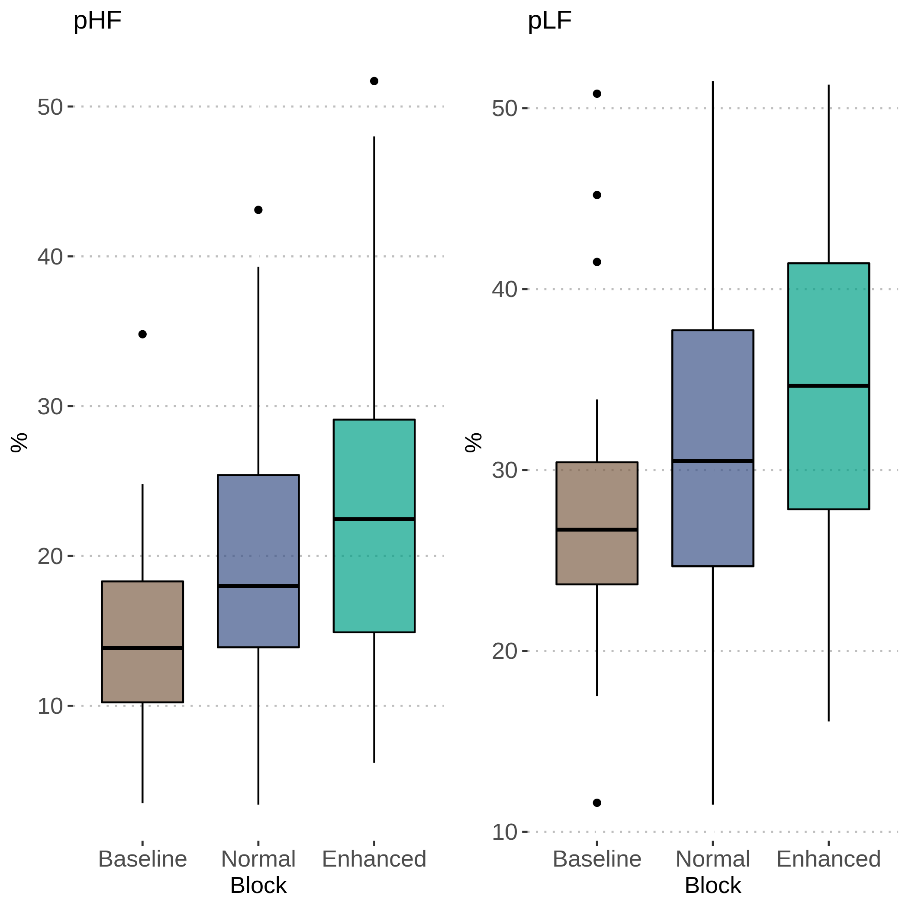


**Figure S7**: Tonic heart rate variability (HRV) measured the percentage of high frequency (pHF) and percentage of low frequency (pLF). Sample size is (n=40) for each all measures except for pHF in the normal block (n=39). Both measures differed from the baseline during both the normal and enhanced stimulation conditions. Horizontal lines in each boxplot represent the median of the corresponding measure while the black dots represent the means. Source data are provided as a Source Data file.

**Supplementary Note 1:**

**Frequency-domain HRV Analysis using low and high frequencies.**

In addition to the time-domain analysis of SDNN, we examined two frequency-domain metrics: the relative power of the low-frequency band (pLF %; 0.04–0.15 Hz), which is generally considered to reflect sympathetic activity, and the relative power of the high-frequency band (pHF %; 0.15–0.40 Hz) which is generally considered to reflect parasympathetic activity.^1^ Both metrics showed significant differences across conditions (F(2,77)=26.64, p<0.0001 for pHF and F(2,78)=10.97, p<0.0001 for pLF). Bonferroni adjusted post-hoc analyses for pHF revealed significant differences between baseline and the normal (paired t-test: t(77)=-5.00, p<0.0001, Cohen d =-1.129) and enhanced conditions (paired t-test: t(77)=-7.1, p<0.0001, Cohen d = -1.587). A similar pattern was observed for Bonferroni adjusted post-hoc analyses of pLF with significant differences between baseline and the normal (paired t-test: t(78)= -2.65, p<0.05, Cohen d =-0.592) and enhanced conditions (paired t-test: t(78)= -4.671, p<0.0001, Cohen d=-1.044).

**Table S2:** Estimated marginal means for the electrograstrogram (EGG), heart rate (HR), heart rate variability (HRV), breathing rate (BR), skin conductance responses (SCR) across tonic (slow – entire block) and phasic (rapid) periods. Source data are provided as a Source Data file.

| Measurement | | Block | Mean | Std. Error | 95% Confidence Interval | |
| --- | --- | --- | --- | --- | --- | --- |
|  |  |  |  |  | Lower Bound | Upper Bound |
| Tonic | **EGG Total Power** | Baseline | 0.00055 | 0.00012 | 0.00031 | 0.00078 |
|  |  | Normal | 0.00067 | 0.00014 | 0.00038 | 0.00096 |
|  |  | Enhanced | 0.00074 | 0.00014 | 0.00047 | 0.00102 |
|  | **HR (Beats Per Minute)** | Baseline | 53.98 | 1.26 | 51.44 | 56.53 |
|  |  | Normal | 58.45 | 1.27 | 55.88 | 61.02 |
|  |  | Enhanced | 59.39 | 1.59 | 56.18 | 62.60 |
|  | **BR (Breaths Per Minute)** | Baseline | 11.52 | 0.34 | 10.83 | 12.21 |
|  |  | Normal | 12.04 | 0.38 | 11.28 | 12.81 |
|  |  | Enhanced | 12.14 | 0.38 | 11.37 | 12.91 |
|  | **HRV (SDNN)** | Baseline | 52.14 | 3.27 | 45.51 | 58.76 |
|  |  | Normal | 61.015 | 3.76 | 53.40 | 68.63 |
|  |  | Enhanced | 64.1 | 4.40 | 55.21 | 72.99 |
| Phasic | **HR (Beats Per Minute)** | Baseline | -0.021 | 0.136 | -0.294 | 0.25 |
|  |  | Normal | 0.789 | 0.18 | 0.42 | 1.16 |
|  |  | Enhanced | 0.835 | 0.193 | 0.44 | 1.23 |
|  | **SCR Maximum value of phasic activity [muS]** | Baseline | -0.057 | 0.051 | -0.159 | 0.045 |
|  |  | Normal | 0.135 | 0.051 | 0.032 | 0.238 |
|  |  | Enhanced | 0.322 | 0.091 | 0.137 | 0.506 |

**Table S3:** Linear mixed effect model results comparing the HRV-SDNN, Phasic HR, Tonic HR, Phasic SCR, and EEG with Sex and Block (i.e., baseline, normal, and enhanced) as fixed factors. Source data are provided as a Source Data file.

| **Analysis** | **Comparison** | **F-Value** | **P-Value** |
| --- | --- | --- | --- |
| HRV-SDNN | ***Sex*** | 0.95 | 0.335 |
|  | ***Block*** | 14.15 | <0.001 |
|  | ***Sex*Block*** | 0.72 | 0.488 |
| Phasic HR | ***Sex*** | 0.31 | 0.583 |
|  | ***Block*** | 8.39 | <0.001 |
|  | ***Sex*Block*** | 0.14 | 0.872 |
| Tonic HR | ***Sex*** | 7.81 | <0.01 |
|  | ***Block*** | 45.38 | <0.01 |
|  | ***Sex*Block*** | 0.58 | 0.560 |
| Tonic BR | ***Sex*** | 0.95 | 0.336 |
|  | ***Block*** | 1.78 | 0.175 |
|  | ***Sex*Block*** | 0.12 | 0.886 |
| Phasic SCR | ***Sex*** | 0.20 | 0.660 |
|  | ***Block*** | 8.21 | <0.001 |
|  | ***Sex*Block*** | 0.69 | 0.504 |
| EGG | ***Sex*** | 0.22 | 0.641 |
|  | ***Block*** | 2.31 | 0.106 |
|  | ***Sex*Block*** | 0.17 | 0.845 |

**Table S4:** Estimated marginal means for the interoceptive sensation ratings. The question was worded as follows: *“How intensely did you feel your stomach or digestive system during the capsule stimulation?”* Participants indicated their response by moving a slider which was anchored on two ends, from 0 *(“Not at all/None”)* to 100 *(“Extremely/The most I have ever felt*”). The other questions followed a similar format, replacing the focus on the relevant organ system (e.g., Heartbeat, Breath, Muscle Tension). Source data are provided as a Source Data file.

| Measurement | Time | Mean | Std. Error | 95% Confidence Interval | |
| --- | --- | --- | --- | --- | --- |
|  |  |  |  | Lower Bound | Upper Bound |
| Stomach/Digestive | Pre- stimulation | 23.55 | 2.95 | 17.57 | 29.53 |
|  | Post- stimulation | 54.22 | 3.57 | 47 | 61.45 |
| Breath | Pre- stimulation | 12.08 | 2.15 | 7.73 | 16.42 |
|  | Post- stimulation | 22.95 | 3.35 | 16.18 | 29.72 |
| Heartbeat | Pre- stimulation | 8.88 | 2.08 | 4.67 | 13.08 |
|  | Post- stimulation | 15.45 | 2.47 | 10.45 | 20.45 |
| Muscle Tension | Pre- stimulation | 17.25 | 2.33 | 12.54 | 21.96 |
|  | Post- stimulation | 14.35 | 2.04 | 10.23 | 18.47 |

**Figure S8:** Spearman correlation between changes (Post – Pre stimulation) in intensity ratings between interoceptive sensations. Changes in stomach/digestive sensation ratings showed a moderate correlation with changes in respiratory sensation ratings and a mild correlation with heartbeat sensation ratings but no correlation with muscle tension ratings. Additionally, changes in respiratory sensation ratings were moderately correlated with heartbeat sensation ratings. **Correlation is significant at the 0.01 level (2-tailed). *Correlation is significant at the 0.05 level (2-tailed). Source data are provided as a Source Data file.

**Table S5:** Separate linear mixed effect model results comparing the stomach/digestive, breath, heartbeat, and muscle tension ratings with Sex and Time (i.e., pre- and post- stimulation) as fixed factors. Source data are provided as a Source Data file.

|  | **Stomach/Digestive** | | **Breath** | | **Heartbeat** | | **Muscle Tension** | |
| --- | --- | --- | --- | --- | --- | --- | --- | --- |
|  | **F-Value** | **P-Value** | **F-Value** | **P-Value** | **F-Value** | **P-Value** | **F-Value** | **P-Value** |
| **Sex** | 0.015 | 0.903 | 0.090 | 0.766 | 0.165 | 0.687 | 1.041 | 0.314 |
| **Time** | 102.132 | <0.001 | 8.915 | 0.005 | 13.393 | <0.001 | 1.265 | 0.268 |
| **Sex*Time** | 2.432 | 0.127 | 4.052 | 0.051 | 2.429 | 0.127 | 0.758 | 0.390 |

**Fig. S9:** Perceptual accuracy measures during the normal and enhanced stimulation blocks based on button presses signifying gut feelings during vibratory stimulation from the capsule. Gray lines present changes in individual performance from the normal to the enhanced block. **A)** Normalized A prime (dashed line shows chance performance based on binomial expansion); **B)** Average response latency (in seconds); and **C)** Standard deviation (STD) of the response latency (in seconds). From left to right panels, the results for the original n=40 sample, original female n=19 sample, female replication n=21 sample, and comparisons between females in the original and replication samples are shown. ***0.01$\leq$ *p* < 0.05, ***p* < 0.01, ****p* < 0.001*;* ns, not significant. Source data are provided as a Source Data file.

**Fig. S10:** Self-reported intensity ratings of different interoceptive sensations experienced before (Pre) and during stimulation (Post; these ratings were provided retrospectively and encompassed sensations experienced during both blocks). **A)** Breath, **B)** Muscle, **C)** Stomach/Digestive, and **D)** Heartbeat tension ratings. Stimulation-induced intensity ratings increased for both stomach and breath sensations. Gray lines show changes in ratings for each individual. From left to right panels, the results for the original n=40 sample, original female n=19 sample, female replication n=21 sample, and comparisons between females in the original and replication samples are shown. *0.01 $\leq$ p < 0.05, **p < 0.01, ***p < 0.001; ns, not significant. Source data are provided as a Source Data file.

**Fig. S11: A)** The positive association between the late ERP signal strength (averaged signal among Cz, CP1, CP2, Pz, POz, O1, Oz, and O2 channels) and perceptual accuracy (normalized A prime) was significant for the normal block (Spearman correlation: *Rho* = 0.523, *p* = 0.040), but not for the enhanced block (Spearman correlation: *Rho* = 0.197, *p* = 0.392). Data from one participant was excluded for being detected as an outlier from the enhanced condition in average ERP amplitude. **B)** A non-significant positive association between late ERP Latency (averaged signal among Cz, CP1, CP2, Pz, POz, O1, Oz, and O2 channels) and response latency was observed for both the enhanced block (Spearman correlation: *Rho* = 0.422, *p* = 0.103), and the normal block (Spearman correlation: *Rho* = 0.393, *p* = 0.078). Shaded areas correspond to the 95% confidence interval for the regressions. Source data are provided as a Source Data file.

**Table S6:** Comparison of perceptual ratings between the original sample (n=40 males and females) and the female replication sample (n=21 females). This table includes 95% effect size estimates for the original and replication samples. Bold font indicates statistically significant p-values. Source data are provided as a Source Data file.

|  | Original sample | | | | Replication sample | | | |
| --- | --- | --- | --- | --- | --- | --- | --- | --- |
| **Parameter** | T-Stats | df | P-Value | Effect Size [confidence interval] | T-Stats | df | P-Value | Effect Size [confidence interval] |
| **A-Prime** | 6.795 | 39 | **<0.001** | 1.070 [0.83 1.45] | 4.806 | 19 | **<0.001** | 1.07 [0.73 1.7] |
| **Latency** | -5.908 | 38 | **<0.001** | -0.946 [-1.51 -0.56] | -3.440 | 15 | **0.004** | -0.860 [-1.98 -0.29] |
| **Latency STD** | -6.915 | 38 | **<0.001** | -1.11 [-1.78 -0.7] | -5.749 | 15 | **<0.001** | -1.44 [-2.49 -0.87] |
| **ERP 400-440** | 2.427 | 36 | **0.020** | 0.399 [0.11 0.71] | 4.058 | 16 | **<0.001** | 0.98 [0.62 1.65] |
| **ERP 440-480** | 4.092 | 37 | **<0.001** | 0.664 [0.36 1.10] | 3.145 | 16 | **0.006** | 0.763 [0.36 1.36] |
| **ERP 480-520** | 4.194 | 36 | **<0.001** | 0.689 [0.38 1.14] | 3.354 | 15 | **0.004** | 0.839 [0.42 1.41] |
| **ERP 520-560** | 5.152 | 37 | **<0.001** | 0.836 [0.57 1.20] | 2.541 | 15 | **0.023** | 0.635 [0.15 1.40] |
| **ERP 560-600** | 3.447 | 37 | **0.001** | 0.559 [0.27 0.87] | 3.650 | 16 | **0.002** | 0.885 [0.49 1.56] |
| **ERP 600-640** | 3.160 | 37 | **0.003** | 0.499 [0.27 0.76] | 2.394 | 15 | **0.030** | 0.598 [0.17 1.07] |
| **ERP 640-680** | 2.997 | 37 | **0.005** | 0.513 [0.26 0.81] | 2.559 | 16 | **0.021** | 0.621 [0.19 1.28] |
| **ERP 680-720** | 1.874 | 38 | **0.069** | 0.300 [0.03 0.60] | 1.390 | 16 | 0.308 | 0.255 [0.22 0.91] |
| **Breath Intensity** | 2.782 | 39 | **0.008** | 0.440 [0.14 0.82] | 1.839 | 20 | 0.081 | 0.401 [-0.02 0.84] |
| **Muscle Intensity** | -1.173 | 39 | 0.248 | -0.186 [-0.55 0.11] | -0.211 | 20 | 0.835 | -0.046 [-0.66 0.35] |
| **Stomach Intensity** | 9.861 | 39 | **<0.001** | 1.56 [1.21 2.13] | 6.569 | 20 | **<0.001** | 1.43 [0.95 2.29] |
| **Heartbeat Intensity** | 3.522 | 39 | **0.001** | 0.557 [0.25 0.88] | 1.474 | 20 | 0.156 | 0.322 [-0.13 0.81] |

**Table S7:** Comparison of peripheral physiological data between the original sample (n=40 males and females) and the female replication sample (n=21 females). This table includes 95% effect size estimates for the original and replication samples. Bold font indicates statistically significant p-values. Source data are provided as a Source Data file.

|  | Original sample | | | | | Replication sample | | | |
| --- | --- | --- | --- | --- | --- | --- | --- | --- | --- |
| Analysis | Comparison | T-Stats | P-Value | Cohen’s d | Effect Size [confidence interval] | T-Stats | P-Value | Cohen’s d | Effect Size [confidence interval] |
| **BR** | Baseline - Enhanced | -1.799 | 0.22767 | -0.406 | [-0.861, 0.049] | 0.204 | 0.83937 | 0.063 | [-0.565, 0.69] |
|  | Baseline - Normal | -1.462 | 0.44340 | -0.33 | [-0.784, 0.124] | -0.055 | 0.95664 | -0.017 | [-0.644, 0.61] |
|  | Normal - Enhanced | -0.34 | 1 | -0.076 | [-0.524, 0.372] | 0.259 | 0.79716 | 0.08 | [-0.548, 0.707] |
| **EGG Total Power** | Baseline - Enhanced | -2.215 | 0.08916 | -0.5 | [-0.955, -0.044] | -2.159 | **0.03709** | -0.678 | [-1.325, -0.032] |
|  | Baseline - Normal | -1.121 | 0.79765 | -0.251 | [-0.699, 0.197] | -2.497 | **0.01688** | -0.785 | [-1.435, -0.134] |
|  | Normal - Enhanced | -1.104 | 0.81894 | -0.249 | [-0.701, 0.203] | 0.336 | 0.73878 | 0.106 | [-0.531, 0.743] |
| **HR Phasic** | Baseline - Enhanced | -3.428 | **0.00293** | -0.772 | [-1.228, -0.316] | -1.176 | 0.24665 | -0.363 | [-0.984, 0.258] |
|  | Baseline - Normal | -3.692 | **0.00124** | -0.825 | [-1.28, -0.371] | -1.525 | 0.13527 | -0.477 | [-1.109, 0.155] |
|  | Normal - Enhanced | 0.238 | 1 | 0.054 | [-0.393, 0.5] | 0.364 | 0.717872 | 0.114 | [-0.512, 0.74] |
| **HR Tonic** | Baseline - Enhanced | -9.018 | **2.99E-13** | -2.016 | [-2.541, -1.492] | -6.032 | **5.13E-07** | -1.896 | [-2.645, -1.146] |
|  | Baseline - Normal | -7.458 | **3.14E-10** | -1.668 | [-2.17, -1.166] | -6.223 | **2.80E-07** | -1.956 | [-2.711, -1.201] |
|  | Normal - Enhanced | -1.56 | 0.36874 | -0.349 | [-0.802, 0.104] | 0.188 | 0.85182 | 0.06 | [-0.603, 0.723] |
| **SCR - Max Phasic** | Baseline - Enhanced | -4.132 | **0.00027** | -0.924 | [-1.375, -0.473] | -2.063 | **0.04585** | -0.645 | [-1.274, -0.017] |
|  | Baseline - Normal | -2.079 | 0.12270 | -0.468 | [-0.915, -0.021] | -0.653 | 0.51784 | -0.204 | [-0.832, 0.423] |
|  | Normal - Enhanced | -2.024 | 0.13925 | -0.456 | [-0.906, -0.006] | -1.395 | 0.17093 | -0.441 | [-1.08, 0.197] |
| **HRV - SDNN** | Baseline - Enhanced | -5.137 | **6.02E-06** | -1.149 | [-1.623, -0.675] | -0.629 | 0.53318 | -0.197 | [-0.836, 0.441] |
|  | Baseline - Normal | -3.812 | **0.00082** | -0.852 | [-1.316, -0.389] | -1.014 | 0.31714 | -0.318 | [-0.959, 0.322] |
|  | Normal - Enhanced | -1.325 | 0.56731 | -0.296 | [-0.747, 0.155] | 0.378 | 0.70740 | 0.121 | [-0.527, 0.769] |


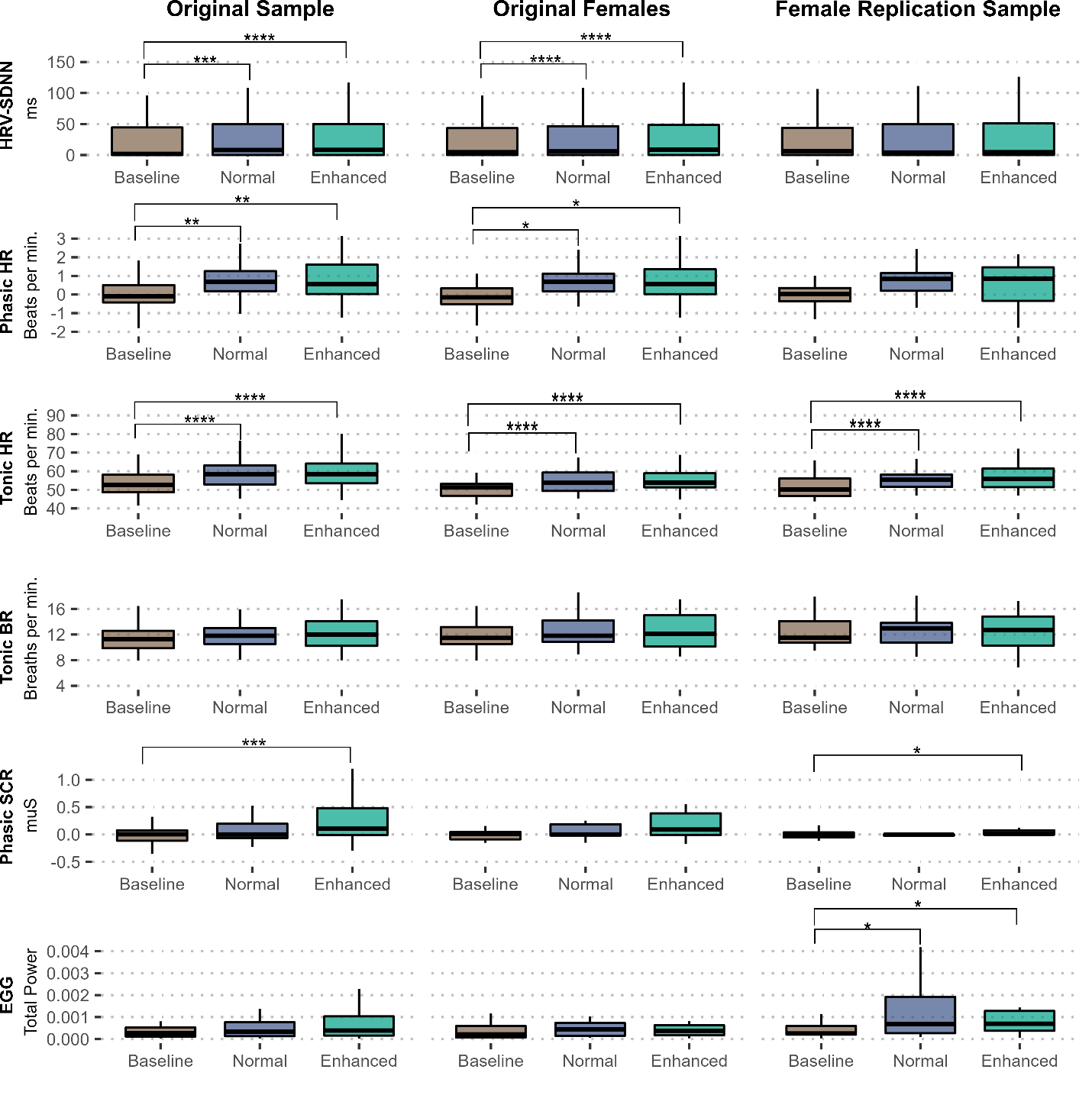


**Fig. S12**: Side-by-side visual illustrations of peripheral physiological measures during phasic (rapid – event-related) and tonic (slow – entire block) periods for the original sample, females from the original sample, and the female replication sample. Horizontal lines in each boxplot represent the median of the corresponding measure. Details on the sample size for each block and dataset are listed in Table S10. **p < 0.01, ****p* < 0.001, *****p* < 0.0001. Source data are provided as a Source Data file.


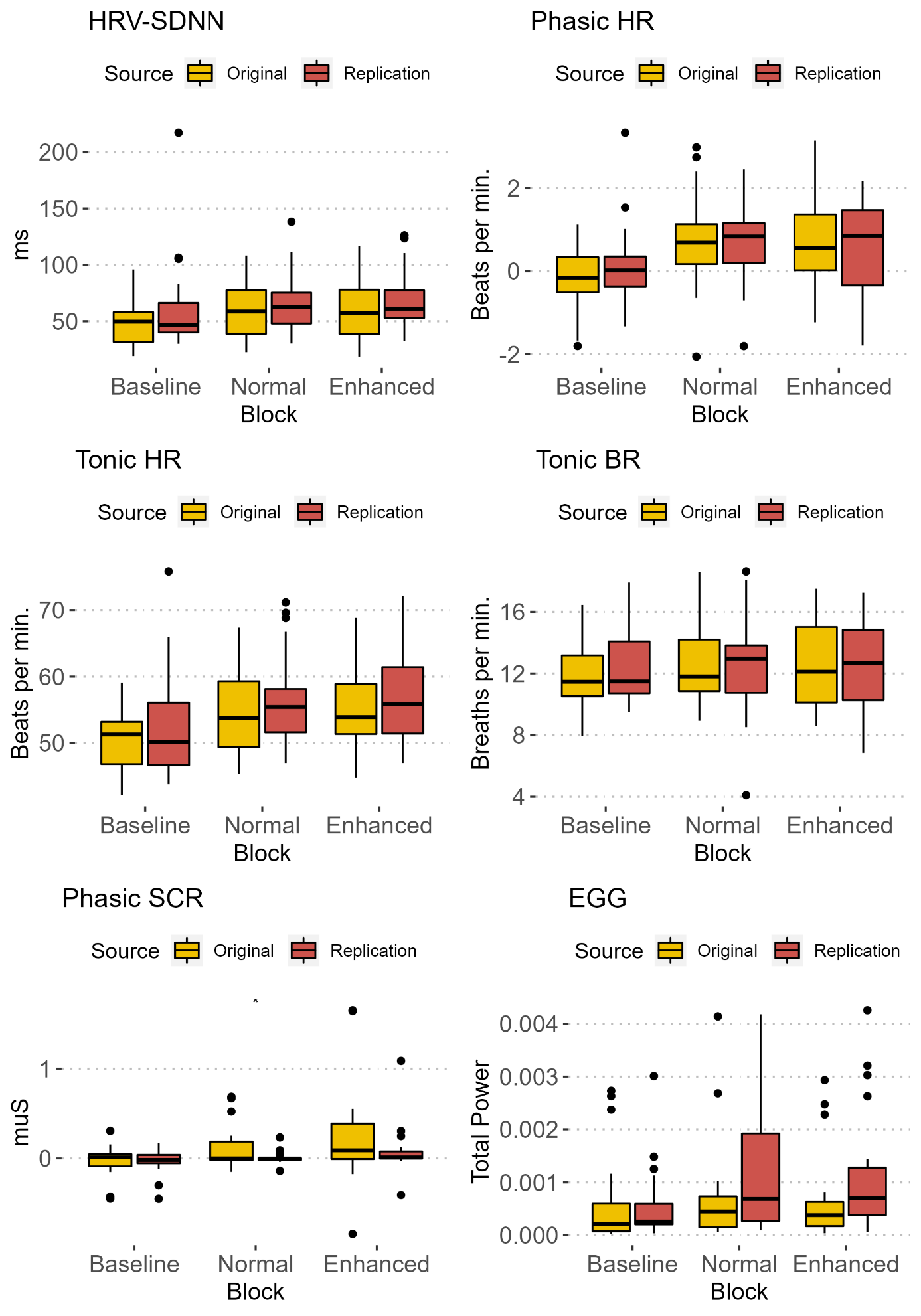


**Fig. S13**: Statistical comparison (independent t-test) of peripheral physiological measures during phasic (rapid – event-related) and tonic (slow – entire block) periods between the females from the original sample and the female replication sample. Sample size for each block in the original sample is (n=19) and replication sample is (n=21) except for the following blocks: 1) Original-EGG-enhanced (n=18), Replication-Phasic HR-normal (n=20), Replication-HRV-SDNN-normal (n=20), Replication-Tonic HR-normal (n=20), Replication-SCR-normal (n=20), Replication-EGG-normal (n=20), Replication-HRV-enhanced (n=20), Replication-Tonic HR-enhanced (n=20), Replication-SCR-enhanced (n=20), and Replication-EGG-enhanced (n=20). There were no significant differences across any of these measures (all p>0.05). Horizontal lines in each boxplot represent the median of the corresponding measure. Source data are provided as a Source Data file.

**Table S8:** Linear mixed effect model results evaluating effect of nonsteroidal anti-inflammatory drugs (NSAIDs) on each perception accuracy, physiological, and subjective measure and their interactions with the block (i.e., normal or enhanced). Source data are provided as a Source Data file.

|  |  |  | Original | | Replication | |
| --- | --- | --- | --- | --- | --- | --- |
| Measurement Type | Variable | Term | F value | P-Value | F-value | P-Value |
| Perceptual Accuracy | Normalized A Prime | Medication | 1.149 | 0.288 | 1.971 | 0.172 |
|  |  | Block | 37.253 | **<0.001** | 4.698 | **0.042** |
|  |  | Medication:Block | 0.305 | 0.583 | 1.531 | 0.231 |
|  | Average Response Latency | Medication | 0.776 | 0.383 | 1.157 | 0.293 |
|  |  | Block | 24.328 | **<0.001** | 7.465 | **0.014** |
|  |  | Medication:Block | 0.668 | 0.419 | 0.352 | 0.56 |
|  | STD Response Latency | Medication | 0.786 | 0.380 | 0.082 | 0.777 |
|  |  | Block | 0.208 | **<0.001** | 10.747 | **0.005** |
|  |  | Medication:Block | 0.162 | 0.690 | 0.26 | 0.617 |
| Physiological Data | SDNN | Medication | 0.062 | 0.804 | 1.124 | 0.294 |
|  |  | Block | 9.37 | **0.003** | 0.445 | 0.509 |
|  |  | Medication:Block | 0.156 | 0.694 | 1.222 | 0.2769 |
|  | HR_Phasic | Medication | 0.037 | 0.848 | 0.089 | 0.767 |
|  |  | Block | 3.294 | 0.073 | 1.381 | 0.245 |
|  |  | Medication:Block | 0.305 | 0.582 | 0.17 | 0.682 |
|  | HR_Tonic | Medication | 1.128 | 0.293 | 0.071 | 0.792 |
|  |  | Block | 28.503 | **<0.001** | 27.448 | **<0.001** |
|  |  | Medication:Block | 0.094 | 0.760 | 0.065 | 0.800 |
|  | BR | Medication | 1.282 | 0.261 | 0.037 | 0.848 |
|  |  | Block | 1.413 | 0.238 | 0.024 | 0.879 |
|  |  | Medication:Block | <0.001 | 0.993 | 1.258 | 0.269 |
|  | Max_Phasic | Medication | 2.036 | 0.157 | 0.019 | 0.892 |
|  |  | Block | 1.241 | 0.269 | 4.342 | **0.044** |
|  |  | Medication:Block | 5.192 | **0.025** | 0.33 | 0.569 |
|  | EGG Total Power | Medication | 0.629 | 0.430 | 0.111 | 0.741 |
|  |  | Block | 0.919 | 0.341 | 5.276 | **0.027** |
|  |  | Medication:Block | 0.303 | 0.584 | 1.976 | 0.168 |
| Self-reported Intensity Ratings | Stomach/Digestive Intensity | Medication | 0.15 | 0.701 | 1.337 | 0.262 |
|  |  | Block | 84.296 | **<0.001** | 16.085 | **0.001** |
|  |  | Medication:Block | 0.04 | 0.842 | 0.392 | 0.539 |
|  | Breath Intensity | Medication | 0.023 | 0.881 | 1.079 | 0.312 |
|  |  | Block | 4.69 | **0.037** | 0.655 | 0.428 |
|  |  | Medication:Block | 1.689 | 0.202 | 0.369 | 0.551 |
|  | Heartbeat Intensity | Medication | 1.164 | 0.287 | 1.739 | 0.203 |
|  |  | Block | 9.248 | **0.004** | 0.79 | 0.385 |
|  |  | Medication:Block | 0.42 | 0.521 | 0.020 | 0.888 |
|  | Muscle Intensity | Medication | 2.814 | 0.110 | 9.067 | **0.005** |
|  |  | Block | 0.109 | 0.745 | 0.956 | 0.759 |
|  |  | Medication:Block | 0.68 | 0.798 | 5.837 | **0.021** |


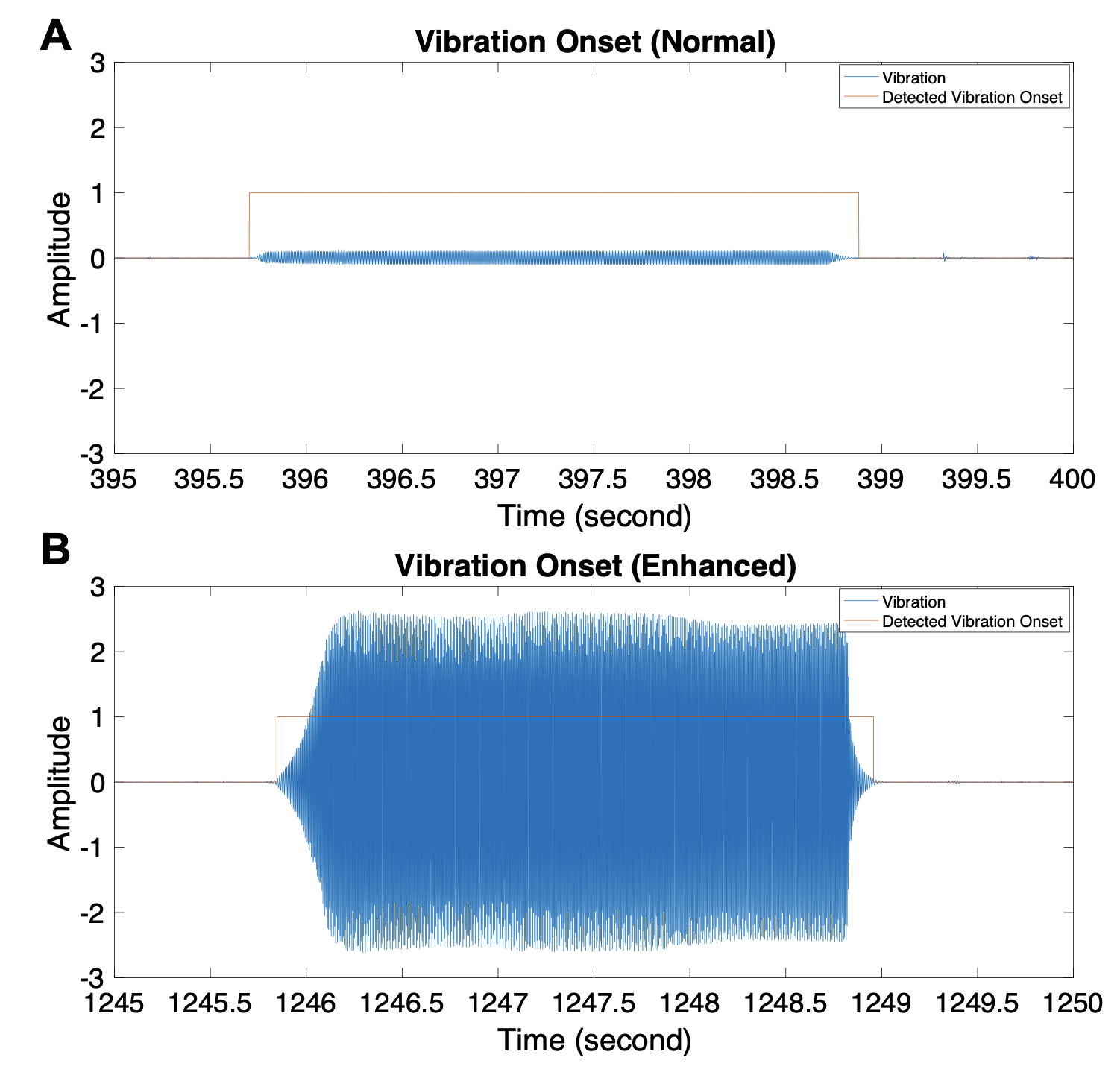


**Figure S14:**Sample digital stethoscope trace for one individual vibration for the normal **(A)** and enhanced **(B)** stimulation conditions. The orange line shows the algorithmically detected timing for the vibration onset as well as offset. Source data are provided as a Source Data file.

**Table S9:** Number of outliers for the perceptual accuracy measures and psychophysiological variables that were identified by removing data points that were three standard deviations (± 3 SD) from the mean before running any statistical analyses. Source data are provided as a Source Data file.

|  | **Original** | | | **Replication** | | |
| --- | --- | --- | --- | --- | --- | --- |
| **Variable** | **Baseline** | **Normal** | **Enhanced** | **Baseline** | **Normal** | **Enhanced** |
| **Normalized A Prime** | NA | 0 | 0 | NA | 1 | 0 |
| **Response Latency** | NA | 0 | 0 | NA | 1 | 0 |
| **STD Response latency** | NA | 0 | 0 | NA | 1 | 0 |
| **ERP 400-440 ms** | NA | 1 | 1 | NA | 0 | 0 |
| **ERP 440-480 ms** | NA | 0 | 1 | NA | 0 | 0 |
| **ERP 480-520 ms** | NA | 0 | 2 | NA | 1 | 0 |
| **ERP 520-560 ms** | NA | 0 | 1 | NA | 1 | 0 |
| **ERP 560-600 ms** | NA | 0 | 1 | NA | 0 | 0 |
| **ERP 600-640 ms** | NA | 0 | 1 | NA | 1 | 0 |
| **ERP 640-680 ms** | NA | 1 | 0 | NA | 0 | 0 |
| **ERP 680-720 ms** | NA | 0 | 0 | NA | 0 | 0 |
| **HRV (SDNN)** | 0 | 0 | 0 | 0 | 1 | 1 |
| **HR (Phasic)** | 0 | 0 | 1 | 0 | 1 | 0 |
| **HR (Tonic)** | 0 | 0 | 0 | 0 | 1 | 1 |
| **BR** | 1 | 0 | 0 | 0 | 0 | 0 |
| **SCR (Maximum value of the phasic activity )** | 0 | 1 | 0 | 0 | 1 | 1 |
| **EGG Total Power** | 0 | 0 | 1 | 0 | 1 | 1 |
| **Normogastria (EGG)** | 0 | 0 | 1 | 0 | 1 | 1 |
| **Tachygastria (EGG)** | 0 | 0 | 1 | 0 | 1 | 1 |
| **Bradygastria (EGG)** | 0 | 0 | 1 | 0 | 1 | 1 |

NA: not applicable

**Table S10:** Number of samples in each block and dataset for peripheral physiological measures. Source data are provided as a Source Data file.

| **Sample** | **Block** | **Sex** | **HRV-SDNN** | **Phasic HR** | **Tonic HR** | **Tonic BR** | **SCR** | **EGG** |
| --- | --- | --- | --- | --- | --- | --- | --- | --- |
| Original | Baseline | F | 19 | 19 | 19 | 19 | 19 | 19 |
| Original | Baseline | M | 21 | 21 | 21 | 20 | 21 | 21 |
| Original | Normal | F | 19 | 19 | 19 | 19 | 19 | 19 |
| Original | Normal | M | 21 | 21 | 21 | 21 | 20 | 21 |
| Original | Enhanced | F | 19 | 19 | 19 | 19 | 19 | 18 |
| Original | Enhanced | M | 21 | 20 | 21 | 21 | 21 | 21 |
| Replication | Baseline | F | 21 | 21 | 21 | 21 | 21 | 21 |
| Replication | Normal | F | 20 | 20 | 20 | 21 | 20 | 20 |
| Replication | Enhanced | F | 20 | 21 | 20 | 21 | 20 | 20 |

* F=Female, M=Male

**
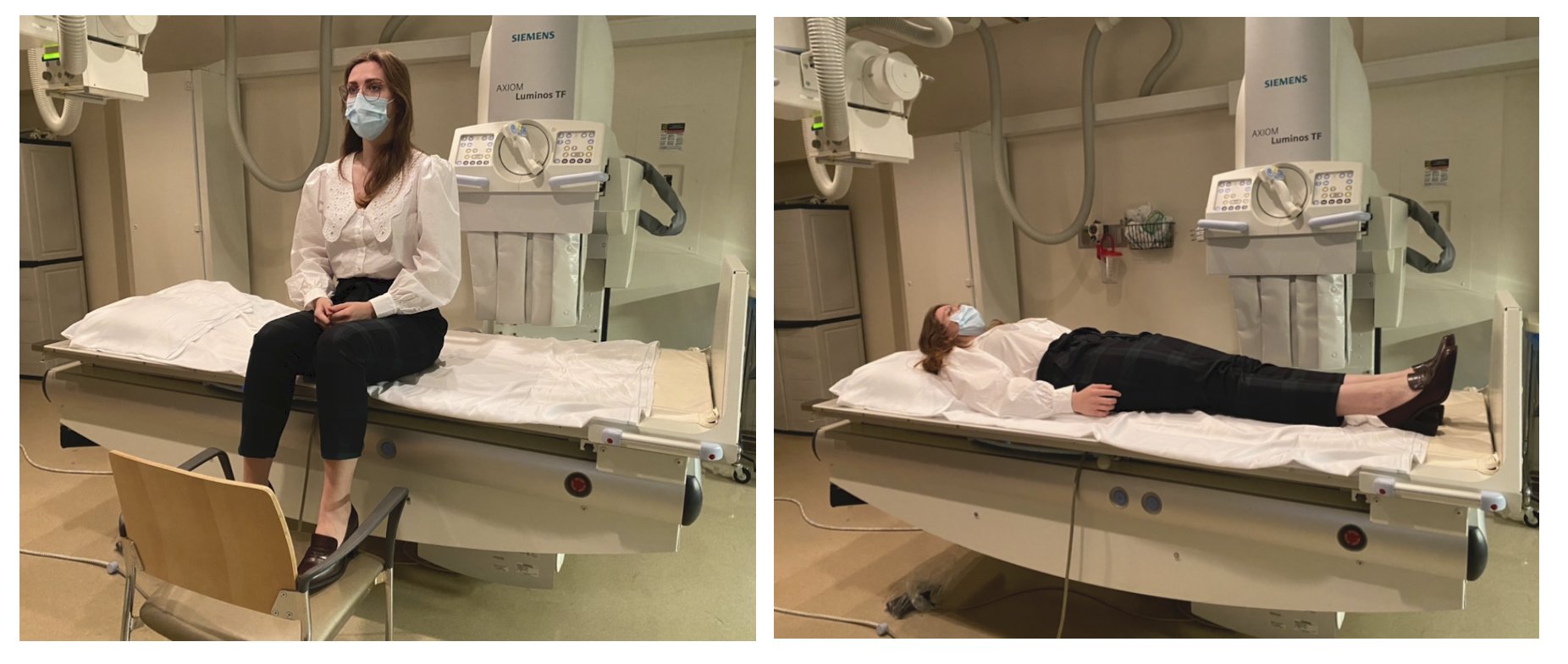
**

**Figure S15.** Abdominal X-ray collection procedure. 10 serial abdominal X-rays were acquired using a Siemens Axiom Luminos TF system, as shown. Prior to capsule ingestion, participants sat upright on the exam table with their feet resting comfortably on a chair to limit abdominal muscle tension and replicate their positioning during the capsule sensation experiment. For each abdominal X-ray, participants briefly turned 90 degrees and then lay supine, returning immediately to the seated position upon completion of the X-ray image. Completion of each transfer and imaging process took approximately one minute, and additional movements were not encouraged throughout the imaging period. The individual shown provided consent for their image to be published.


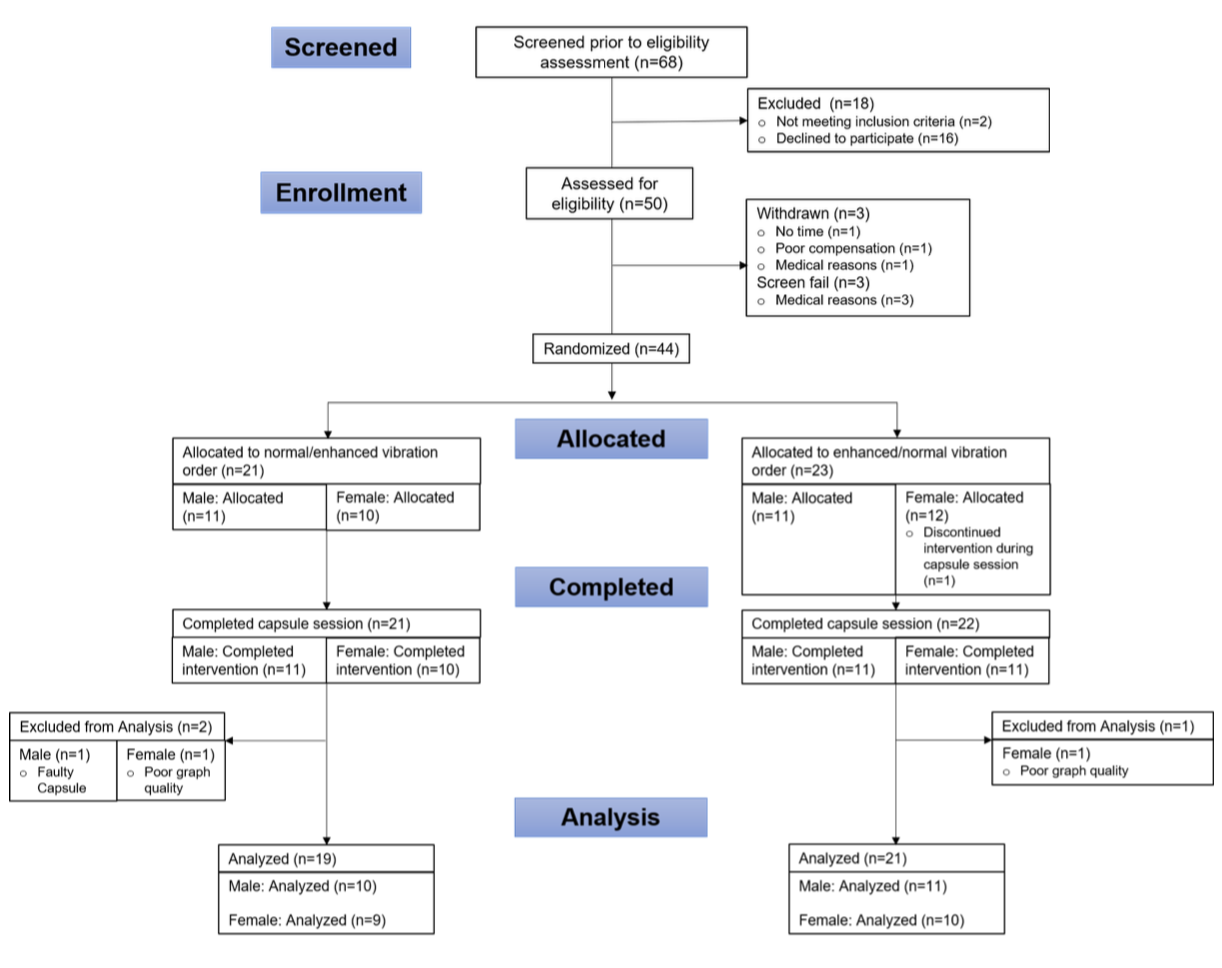


**Figure S16.** Diagram showing the flow of study participants for the original experiment (n=40 participants).

**
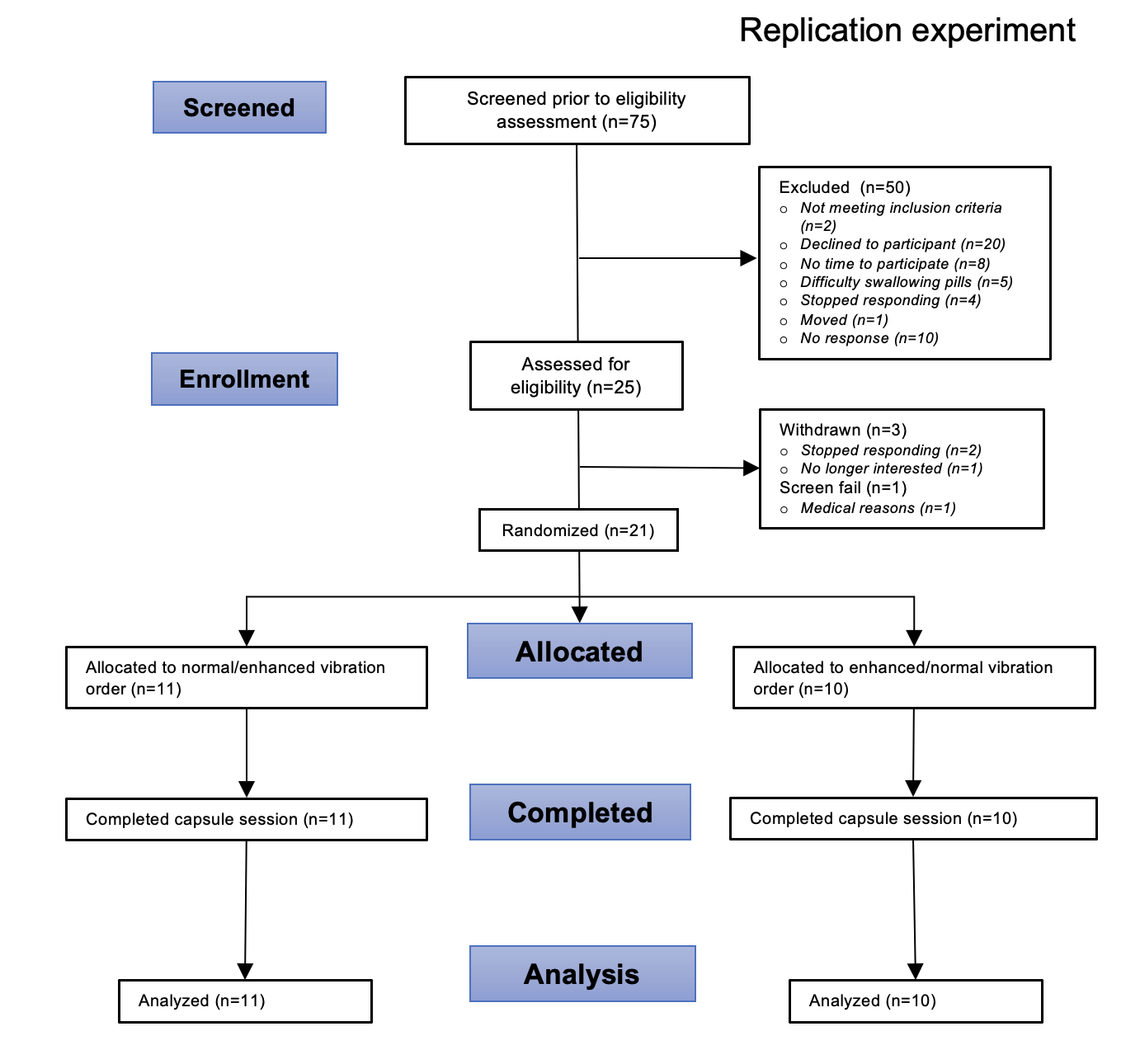
**

**Figure S17.** Diagram showing the flow of study participants for the replication experiment (n=21 female participants).

**Supplementary Reference**

1. Shaffer F, Ginsberg JP. An Overview of Heart Rate Variability Metrics and Norms. *Front Public Health* 5, 258 (2017).
